# Worldwide Incidence of Ocular Melanoma and Correlation With Pigmentation-Related Risk Factors

**DOI:** 10.1167/iovs.64.13.45

**Published:** 2023-10-30

**Authors:** Mike Wu, Serdar Yavuzyiğitoğlu, Erwin Brosens, Wishal D. Ramdas, Emine Kiliç, on behalf of the Rotterdam Ocular Melanoma Study Group (ROMS)

**Affiliations:** 1 Department of Ophthalmology, Erasmus MC University Medical Center, Rotterdam, The Netherlands; 2 Department of Clinical Genetics, Erasmus MC University Medical Center, Rotterdam, The Netherlands; 3 Erasmus MC Cancer Institute, Erasmus MC University Medical Center, Rotterdam, The Netherlands

**Keywords:** ocular melanoma (OM), uveal melanoma (UM), iris color, eye color, pigmentation, risk factor, incidence, allele frequency, global patterns, trends, cancer

## Abstract

**Purpose:**

The worldwide incidence of ocular melanoma (OM), uveal melanoma (UM), and conjunctival melanoma has last been reported on 15 years ago. Recently, light iris color and four specific single-nucleotide-polymorphisms (SNPs) have been identified as a UM-risk factor. Furthermore, six iris color predicting SNPs have been discovered (IrisPlex). Interestingly, two of these (rs129138329 and rs12203592) are also UM-risk factors. We collected worldwide incidence data of OM and investigated its correlations with iris color, IrisPlex SNPs, and UM-risk SNPs.

**Methods:**

Cases of OM, as defined by the International Classification of Diseases Oncology C69 (eye), 8720/3 to 8790/3 (malignant melanoma), and 8000 to 8005 (malignant neoplasm), between 1988 and 2012, were extracted from the Cancer Incidence in Five Continents. Incidence rates were age-standardized and their trends were analyzed with joinpoint regression and age period cohort modeling. Frequencies for each country of iris color, IrisPlex SNPs, and UM-risk SNPs were collected from the literature.

**Results:**

Incidence rates were generally ≥8.0 cases per million person-years in Northern Europe, Western Europe, and Oceania; 2.0 to 7.9 in North America, Eastern Europe, and Southern Europe; and <2.0 in South America, Asia, and Africa. OM incidence correlated with latitude (*r* = 0.77, *P* ≤ 0.001) and is expressed as a north-to-south decreasing gradient in Europe. SNP rs12913832 correlated with OM incidence (*r* = 0.83, *P* ≤ 0.001), blue iris color (*r* = 0.56, *P* ≤ 0.05), green iris color (*r* = 0.51, *P* ≤ 0.05), and brown iris color (*r* = −0.64, *P* ≤ 0.01). Trends were stable for most countries (28/35).

**Conclusions:**

OM incidence is highest in populations of European ancestry and lowest in populations of Asian and African ancestry. Overall, trends are stable, and the spatial correlation among OM incidence, iris color, and rs12913832 may support the role of pigmentation-related risk factors in OM development.

Ocular melanoma (OM) can be divided into uveal and non-uveal melanoma. Uveal melanoma (UM) comprises the larger group of OMs (85.0%)^1^ and consists of choroidal, ciliary body, and iris melanoma. Non-uveal melanoma includes conjunctival melanoma (4.8%) and ocular melanoma from other sites (10.2%).^1^ Several pigmentation-related risk factors are associated with UM, including light iris color,^2^ fair skin color,^2^ ability to tan,^2^ atypical cutaneous nevi,^3^ common cutaneous nevi,^3^ cutaneous freckles,^3^ and iris nevi.^3^ Evidence for an association between ultraviolet light and development of uveal melanoma is inconsistent.^4^ However, the latest publications have not found a ultraviolet (UV)-radiation signature in choroidal or ciliary body melanoma,^5^^,^^6^ in contrast with iris melanoma^6^ and conjunctival melanoma.^7^ Additional risk factors for conjunctival melanoma include conjunctival naevi^8^ and conjunctival primary acquired melanosis.^9^ More recently, several single-nucleotide-polymorphisms (SNPs) have been associated with UM risk, including rs3759710 (chr14:89955214, TDP1),^10^ rs421284 (chr5:1325475, CLPTM1L),^11^^,^^12^ rs12913832 (chr15:28120472, HERC2),^12^^,^^13^ and rs12203592 (chr6:396321, IRF4).^12^^,^^13^ Interestingly, the latter two SNPs (rs12913832 and rs12203592) have also been identified as IrisPlex SNPs, which are linked to iris color.^14^ In addition, there are 4 more IrisPlex SNPs which are solely associated with iris color, specifically rs1800407 (chr15:27985172, OCA2), rs12896399 (chr14:92307319, LOC105370627), rs16891982 (chr5:33951588, SLC45A2), and rs1393350 (chr11:89277878, TYR).^14^

The incidence of UM shows marked geographic variation, ranging from less than 1 case per million person-years in Asia^15^^–^^18^ to exceeding 10 per million in Northern Europe.^15^^,^^19^^–^^22^ In the United States,^23^ Canada,^24^ Australia,^25^ and the other European regions,^15^^,^^19^^,^^24^^,^^26^^,^^27^ an average of six cases per million was observed. Nationwide studies have reported a stable incidence of UM in Sweden,^22^ Germany,^27^ and the United States,^23^ whereas Denmark,^21^ South Korea,^17^ and Canada^24^ experienced an increase. For conjunctival melanoma, the incidence remained stable at 0.46 cases per million person-years, ranging from 0.28 in Eastern Europe to 0.92 cases per million in Northern Europe.^28^ Most nationwide studies also reported stable incidence rates for conjunctival melanoma in Canada,^29^ Poland,^30^ and Denmark.^31^ A significant increase was reported in Sweden^32^ and the United States,^33^ where the incidence also differed among ethnic groups.^34^

Since the 1960s, the International Agency for Research on Cancer (IARC) and the International Association of Cancer Registries (IACR) have been collecting data from cancer registries worldwide and publishing the aggregated data as the Cancer Incidence in Five Continents (CI5) series. Volumes VII, VIII, IX, X, and XI of the CI5 series contain incidence and population-at-risk data from 150, 186, 225, 290, and 343 registries, respectively. The data are presented in 5-year periods and includes information from 50, 57, 60, 68, and 65 countries for the time periods of 1988 to 1992, 1993 to 1997, 1998 to 2002, 2002 to 2007, and 2008 to 2012, respectively.^35^^–^^39^ Additionally, the IARC and IACR publish the Cancer Incidence in Five Continents Time Trends (CI5Plus), which provides updated annual incidence data from 106 cancer registries. These registries have histological data available for a minimum of 15 consecutive years, as published in the CI5 reports.^35^^–^^39^ However, the most recent reports on worldwide UM incidence were published over 15 years ago, covering only the period before 1997.^15^^,^^19^ Because it was not possible to distinguish melanoma between ocular sites, our aim was to determine the worldwide incidence and its changes over time specifically for OM in general from 1988 until the latest available data. Furthermore, we investigated the spatial distribution among OM incidence, iris color, and SNPs associated with iris color and/or UM risk.

## Methods

### Definition of Cases

Cases of OM were defined by the International Classification of Diseases Oncology, Third Edition (ICD-O-3) topography code C69 (eye) and morphology code 8720/3 to 8790/3 (malignant melanoma).^40^ All cases from CI5Plus and CI5 series were microscopically verified. To align with the trend of microscopic verification,^1^^,^^15^^,^^19^^,^^23^ we re-allocated unspecified cases of malignant neoplasms to OM, as was previously done by Stang et al.^15^ These unspecified cases are defined as malignant neoplasms in the eye, ICD-O-3 codes C69 and 8000 to 8005 (malignant neoplasm), for individuals aged 15 years and older, and were considered as non-microscopically verified cases. The re-allocation was only performed in North America, Europe, and Asia, as previously described.^15^ To maintain consistency with earlier reports from Africa and South America, that had a 100% microscopic verification rate, we did not re-allocate cases from these regions.^41^^,^^42^ OM cases of unknown age of onset (*n* = 36) were excluded.

### OM Incidence

Incidence data was extracted from the CI5plus^35^^–^^39^ and CI5 volumes.^35^^–^^39^ The incidence and population data from the CI5Plus were aggregated into the same 5-year periods as the CI5 volumes. Duplicate data and overlapping populations were excluded. The corresponding populations-at-risk were obtained from the CI5Plus population pyramids.^43^ A more detailed description on the inclusion criteria and coverage of the CI5 registries is provided by Bray et al.^44^^,^^45^ Most countries had one cancer registry covering the entire country, but if more than one registry was available, the data of individual cancer registries was aggregated (Supplementary Table S1). Countries were further grouped into geographic regions according to the United Nations Statistics Division,^46^ except for Eastern Africa, Western Africa, and Southern Africa, which we pooled into Sub-Saharan Africa, due to a small number of cancer registries in these regions. Furthermore, we categorized countries from North America, Europe, and Oceania, that have populations of predominantly European ancestry, as Western countries, whereas those from Asia, South America (due to genetic admixture), and Africa as non-Western countries.

### Statistical Analysis

We estimated age-standardized incidence rates (ASRs) per million person-years using the standard population of the respective continents whenever available. In populations where continent-specific standard populations were not available, the World Standard population was used (see Supplementary Table S1). To achieve this, incidence rates were standardized by the European,^47^ United States,^48^ Australian,^49^ and World Health Organization (WHO) 2000 to 2025^47^ standard populations. We estimated corresponding 95% confidence intervals using Fay and Feuer's gamma interval approximation with Tiwari's modification.^50^ At least 10 cases were required for ASR estimation.^51^ Temporal trends of countries with a minimum of 20 years of follow-up were analyzed via the average annual percentage change (AAPC) of the linear regression model obtained through joinpoint regression analysis, using Joinpoint Regression Program version 4.9.0.0 (Statistical Research and Applications Branch, National Cancer Institute). To investigate significant AAPCs effects, an age period cohort (APC) analysis was performed to identify age, period, and cohort effects. This analysis was conducted using the R package “apc” version 2.0.0 within R version 4.2.1. Age specific incidence and population pyramids were investigated to assess the influence of life expectancy on incidence. Choropleth maps were created using Microsoft Excel for Microsoft 365 MSO, version 2110 Build 16.0.14527.20234, and Adobe Illustrator, version 26.1 64-bit. Statistical analysis and graph computations were performed with IBM SPSS Statistics for Windows, version 26.0. Estimated incidence rates were validated with R package “epitools” version 0.5–9.

### Literature Search on Iris Color and SNPs

A literature search was performed to gather information on the geographic distribution of iris color (Supplementary Fig. S1). Iris color was categorized in blue and grey (hereafter called blue), green and intermediate (hereafter called green), and brown and hazel (hereafter called brown). When multiple frequencies of iris color were available within a population, a weighted average was estimated. The allele frequencies of 4 UM risk SNPs and 6 IrisPlex SNPs, of which 2 SNPs are overlapping, specifically rs3759710 (UM risk),^10^ rs421284 (UM risk),^10^^,^^12^ rs12913832 (UM risk and IrisPlex),^12^ rs12203592 (UM risk and IrisPlex),^12^ rs1800407 (IrisPlex),^14^ rs12896399 (IrisPlex),^14^ rs16891982 (IrisPlex),^14^ and rs1393350 (IrisPlex),^14^ were extracted from the 1000 Genomes Project^52^ (1KGP) phase III dataset via Ensembl genome browser (version 109, genome assembly GRCh38.p13). Populations from 1KGP were matched with cancer registries from CI5Plus and CI5 volumes based on topographic location and ethnicity, if available. For instance, the 1KGP population named “People with African Ancestry in Southwest USA” was matched with cancer registries from the United States covering African American populations (see Supplementary Table S1). Latitude, longitude, and exposure to solar UV radiation data by country were extracted from Google's Geocoding API^53^ and the WHO's Global Health Observatory.^54^ Choropleth maps, scatter plots, and Spearman's correlation coefficients were generated to investigate the spatial distribution of OM incidence, the absolute values of latitude, longitude, UV radiation, frequency of iris colors, and allele frequencies of SNPs of interest. Correlation matrices with Spearman's correlation coefficient were plotted using R package “psych” version 2.3.6. Sensitivity analyses were conducted to rule out potential data biases.

## Results

### Worldwide Incidence From 1988 to 2012

A total of 347 cancer registries covering 360 populations in 80 countries from 1988 to 2012 were included in the analysis. Among these, 48 countries had one registry, 7 had 2 registries, and the remaining 25 countries had 3 or more registries (see Supplementary Table S1, Supplementary Fig. S2). This resulted in a total number of 91,738 cases of OM (Supplementary Table S2). Of these, Europe accounted for 49,029 cases of OM, gathered from 155 cancer registries; North America registered 34,455 cases from 14 registries, and Oceania had 5,008 cases from 10 registries. The remaining 3246 cases of OM were reported from the rest of the world, obtained from 168 registries. Despite the worldwide coverage, 96.5% of the OM cases originated from Western countries with predominantly White populations of European ancestry.

**Figure 1.**
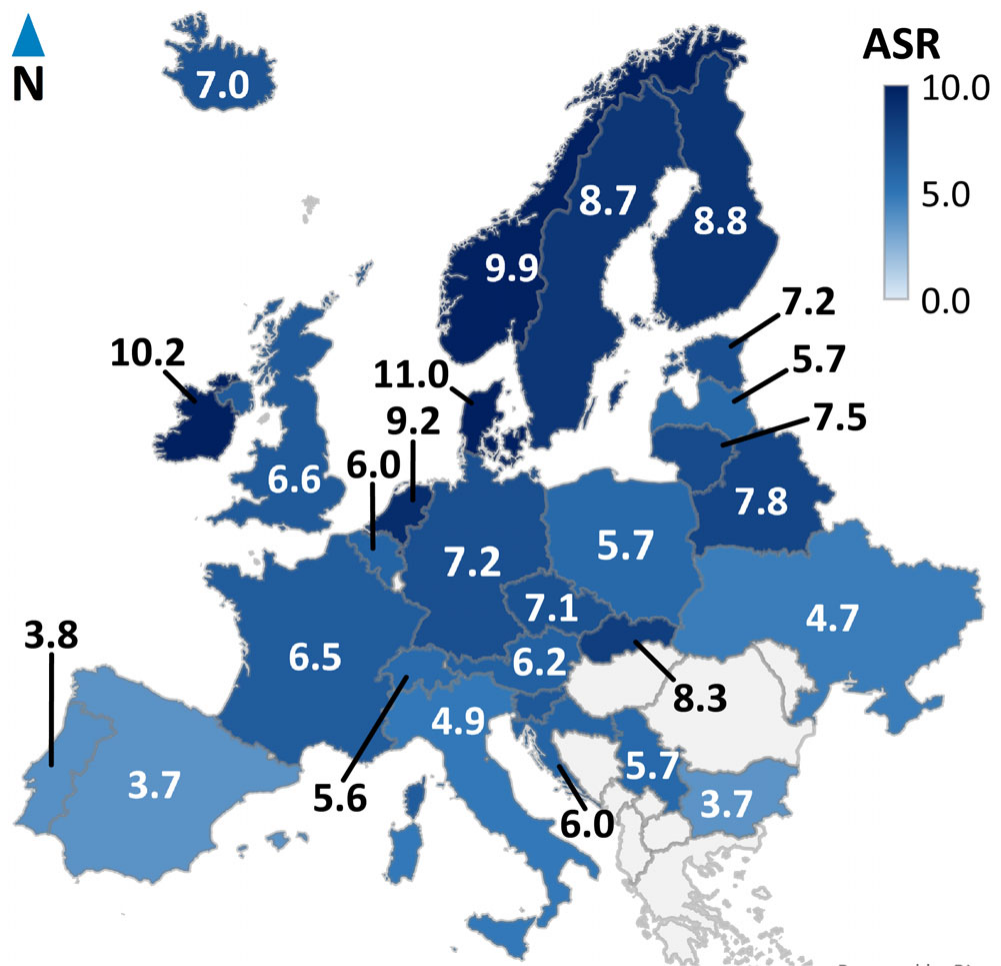
Choropleth map of age-standardized incidence rates (ASR) of ocular melanoma in Europe. The age-standardized incidence rates (ASR) of ocular melanoma in Europe is highest in countries around the Baltic Sea and North Sea, specifically Denmark (ASR: 11.0 cases per million person-years), Ireland (10.2), Norway (9.9), the Netherlands (9.2), Finland (8.8), and Sweden (8.7). High intermediate ASRs are observed in Slovakia (ASR: 8.3 cases per million person-years), Belarus (7.8), Lithuania (7.5), Estonia (7.2), Germany (7.2), the Czech Republic (7.1), Iceland (7.0), the United Kingdom (6.6), and France (6.5). Low intermediate ASRs are observed in Austria (ASR: 6.2 cases per million person-years), Croatia (6.0), Poland (5.7), Latvia (5.7), and Switzerland (5.6). Low ASRs are observed in Italy (ASR: 4.9 cases per million person-years), Portugal (3.8), Spain (3.7), and Bulgaria (3.7).

**Figure 2.**
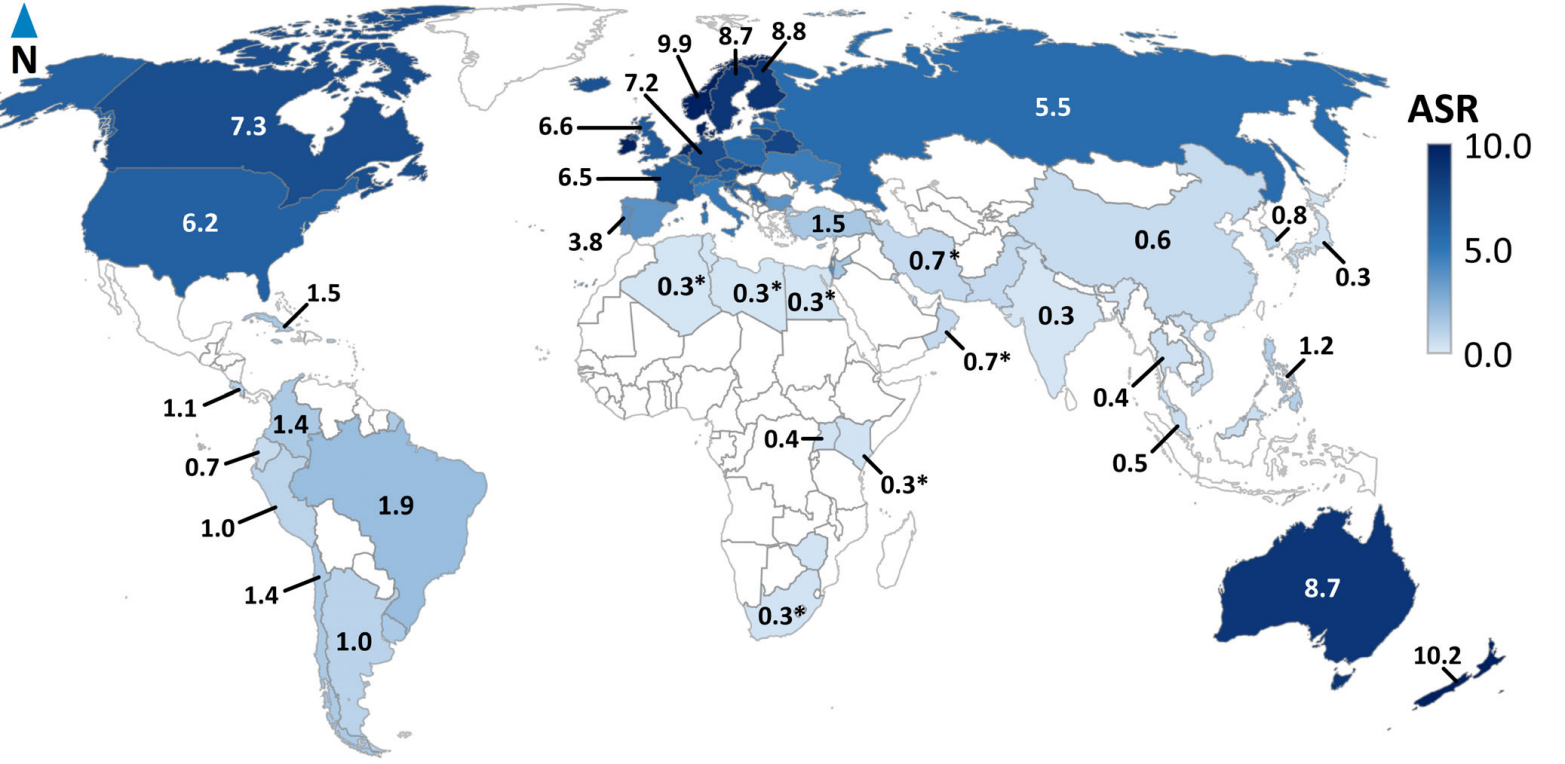
Choropleth map of age-standardized incidence rates (ASR) of ocular melanoma worldwide, 1988 to 2012. The age-standardized incidence rates (ASR) of ocular melanoma are highest in Western countries, specifically Australia (ASR: 8.7 cases per million person-years), New Zealand (10.2), United States (6.2), Canada (7.3), and European countries (lowest ASR: 3.7 in Southern Europe and highest ASR: 11.0 in Northern Europe). * Mean ASR from corresponding region presented due to <10 cases per country.

Consequently, the highest ASRs were observed in Northern and Western Europe, including Denmark (ASR: 11.0 cases per million person-years), Ireland (10.2), Norway (9.9), the Netherlands (9.2), Finland (8.8), and Sweden (8.7). Oceania, represented by Australia (ASR: 8.7 cases per million person-years) and New Zealand (10.2), had similarly high ASRs (Figs. 1, 2). High intermediate ASRs are observed in North America, including Canada (ASR: 7.3 cases per million person-years) and the United States (6.2), as well as various Eastern and Western European countries. In contrast, most Southern European countries had ASRs between two and six cases per million person-years, whereas South America, Asia, and Africa exhibited the lowest incidence rates (ASR < 2.0). Interestingly, we discovered significant differences in ASR within the ethnic subpopulations of the United States. Specifically, the ASR for White Americans was 7.13 cases per million person-years, for Asian Americans it was 0.96, and for African Americans it was 0.63. These ASR values align with the continents of genetic ancestry, with Europe having an ASR of 6.62, Asia with 0.72, and Africa with 0.30 cases per million person-years (see Supplementary Table S2).

Along with OM incidence, we plotted additional data on latitude, longitude, and UV radiation in a correlation matrix (Supplementary Fig. S3A). The distribution of ASR exhibited a significant positive correlation with latitude, indicating that higher latitudes were associated with higher ASR. Western countries typically clustered between latitudes 40 and 60, with ASR ranging from 4 to 10 cases per million person-years. However, several outliers from Asian and South American countries, were situated near latitudes 40 or below 10, with ASR below 2 cases per million person-years (see Supplementary Fig. S3A). Nevertheless, a north-to-south decreasing latitude gradient was observed in Europe (see Fig. 1). Latitude is geographically correlated with UV radiation, and, in our analyses, we observed a correlation between ASR and UV radiation as well. For the sensitivity analysis of these correlations, Western countries (marked with § in Supplementary Table S2) and non-Western countries (marked with ∥ in Supplementary Table S2) were selected in a 1:1 ratio. The positive correlation between ASR and latitude (*r* = 0.77, *P* ≤ 0.001) and the negative correlations between ASR and UV radiation (*r* = −0.75, *P* ≤ 0.001), as well as between latitude and UV radiation (*r* = −0.95, *P* ≤ 0.001), remained significant (see Supplementary Fig. S3B).

### Age-Specific Incidence and Population Pyramids

The age-specific incidence of OM follows a J-curve, with the highest peak-incidence occurring in the 80 to 84 years age group for Oceania, Europe, and North America. However, the J-curve pattern is less noticeable in Asia and South America, and completely absent in Africa (Supplementary Fig. S4). These continental differences in age-specific incidence may be attributed to the age distributions of the respective populations. An aging population pyramid is observed in Europe (Supplementary Fig. S5A), Oceania (Supplementary Fig. S5B), and North America (Supplementary Fig. S5C), whereas a growing population pyramid is evident in Africa (Supplementary Fig. S5F). The population pyramids of South America (Supplementary Fig. S5D) and Asia (Supplementary Fig. S5E) exhibit characteristics of both an aging and a growing population. As a result, aging populations have a higher number of registered cases of ocular melanoma compared to growing populations.

### Temporal Trends in Incidence

ASRs of 35 counties by 5-year periods are displayed in a heatmap and their temporal trends were analyzed using Joinpoint regression and APC analysis (see the Table). The highest recorded incidence occurred in Denmark, with 12.1 cases per million person-years during the 2003 to 2007 period, followed by 11.3 in the preceding and subsequent 5-year periods. Northern and Western European countries, Australia, and New Zealand clustered together with ASRs above 8.0, whereas Canada and the United States generally displayed ASRs between 6.0 and 7.9 cases per million person-years. Intermediate ASRs were observed in Eastern and Southern Europe, followed by ASRs below 1.0 cases per million person-years in non-Western countries. Out of the 35 analyzed countries, only 7 (20%) experienced a change in incidence. Ireland (AAPC = −0.8, *P* ≤ 0.05), India (AAPC = −5.6, *P* ≤ 0.05), and Finland (AAPC = −1.9, *P* ≤ 0.05) exhibited a decrease in incidence, whereas South Korea (AAPC = 1.8, *P* ≤ 0.05), Sweden (AAPC = 1.0, *P* ≤ 0.05), and Russia (AAPC = 4.9, *P* ≤ 0.05) experienced an increase. Age-cohort effects could explain only the significant changes observed in Ireland and Sweden (see the Table).

**Table.** Heatmap, Trend, and Age Period Cohort Analysis of Ocular Melanoma Incidence

	
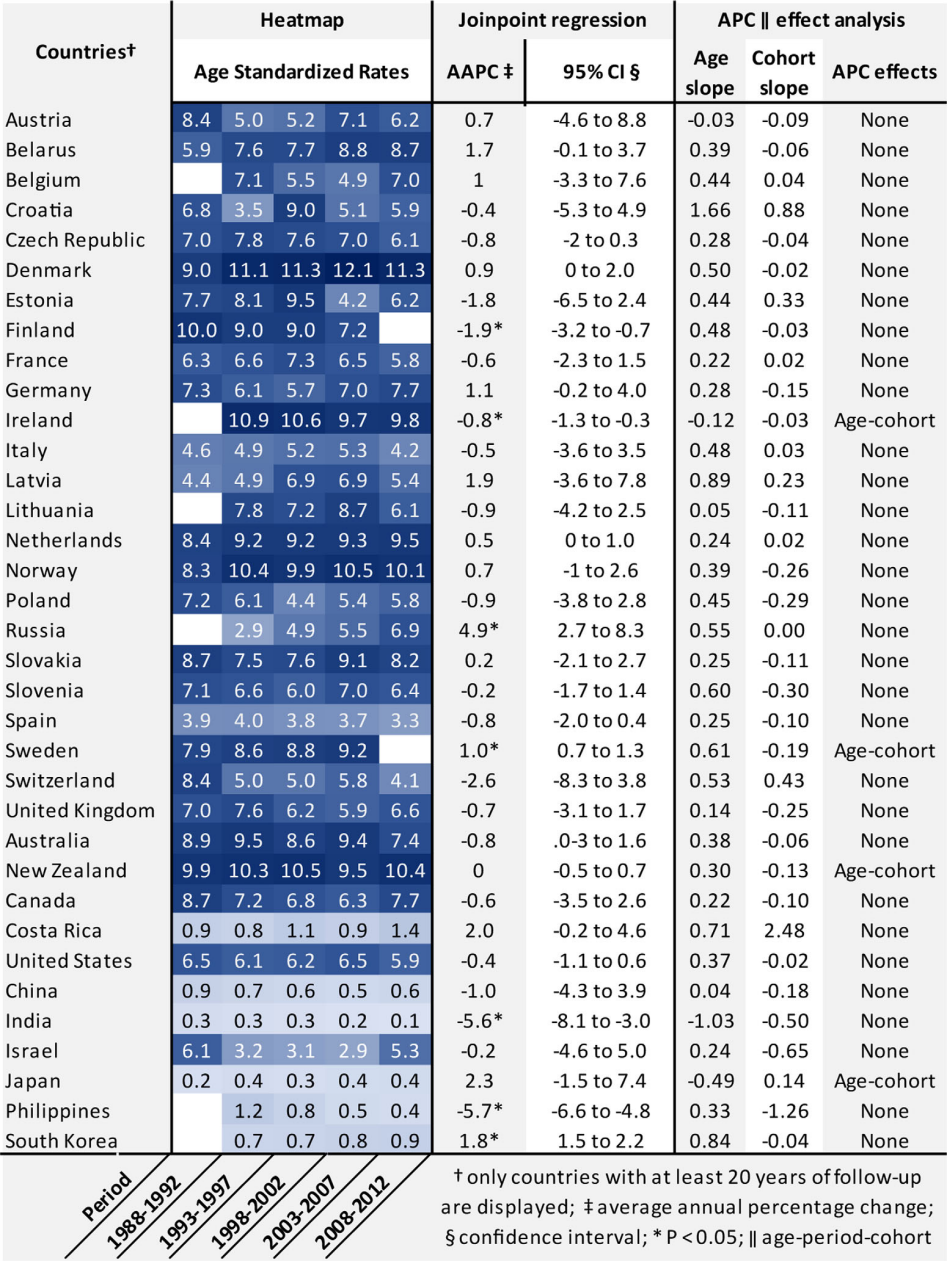	

The age-adjusted incidence rates (ASR) of selected countries by 5-year periods are clustered by continent. Higher ASRs > 6.0 cases per million person-years are clustered in countries of Northern and Western Europe, Australia, New Zealand, Canada, and the United States, whereas lower ASRs < 1.5 are clustered in non-Western countries, specifically Costa Rica, South Korea, the Philippines, China, Japan, and India. Most countries displayed stable trends in incidence, except for 7 out of 35 (20%) analyzed countries. Ireland, Finland, India, and the Philippines experienced a significant decrease in incidence, whereas Sweden, Russia, and South Korea showed a significant increase. Only the changes in Ireland and Sweden could be explained by age-cohort effects via APC analysis. No significant APC effects were found in other cohorts.

### Distribution of Iris Color, IrisPlex, and UM Risk SNPs

We gathered the allele frequencies for the 8 specific SNPs of interest from all 26 populations of the 1KGP (Supplementary Table S3). Among the examined SNPs, we spatially visualized the distribution of allele frequencies of SNP rs12913832, which is the most important predictor for iris color^14^ (Supplementary Fig. S6). We were able to match 20 out of 26 populations with the cancer registries in our dataset (see Supplementary Table S1), whereas 6 populations were excluded (see Supplementary Table S3). In addition, we collected the iris color frequencies, specifically blue, green, and brown, from 18 references covering 35 countries (Supplementary Table S4). Data from ocular melanoma incidence, iris color frequencies, and SNPs of interest were matched to create a correlation matrix (Supplementary Fig. S7).

In this correlation matrix, rs12913832 exhibited a strong correlation not only with the distribution of ASR (*r* = 0.83, *P* ≤ 0.001), but also with the distribution of blue iris color (*r* = 0.56, *P* ≤ 0.05), green iris color (*r* = 0.51, *P* ≤ 0.05), and brown iris color (*r* = −0.64, *P* ≤ 0.01). Furthermore, rs12913832 showed significant correlations with IrisPlex and UM risk SNPs as well, including rs421284 (*r* = 0.51, *P* ≤ 0.05), rs1393350 (*r* = 0.90, *P* ≤ 0.001), rs1800407 (*r* = 0.76, *P* ≤ 0.001), rs3759710 (*r* = −0.55, *P* ≤ 0.05), rs1220592 (*r* = 0.87, *P* ≤ 0.001), and rs16891982 (*r* = 0.97, *P* ≤ 0.001). Consequently, all of these SNPs, except for rs421284, displayed significant correlations with ASR (see Supplementary Fig. S7).

For the sensitivity analysis of these correlations, cancer registries were matched in a 1:1 ratio across different continents, including Europe, South America, Africa, and Asia. Considering the substantial variations in ASR within Europe, we further divided European cancer registries into three subgroups representing a higher, medium, and lower incidence cohort (Supplementary Table S5). The Spearman's correlation coefficients between ASR and SNPs remained consistent across all three subgroups. Notably, the correlation coefficients between ASR and all three iris colors increased in the lower, medium, and higher incidence cohort (Supplementary Table S6).

## Discussion

In this study, we described the worldwide incidence of OM, spanning from 1988 to the latest available data, which is 2012 as of the time of writing. Our dataset predominantly included cases from Western countries with primarily White populations of European ancestry (see Supplementary Table S2). The highest ASRs were observed in Western countries, such as Denmark, Ireland, New Zealand, and Norway, whereas the lowest incidence rates were observed in South America, Africa, and Asia (see Figs. 1, 2). Notably, OM incidence among ethnic populations in the United States is comparable to that in their respective continents of origin (see Supplementary Table S2).

Interestingly, even though OM incidence was significantly correlated with latitude, outliers from Asia and South America contradicted the expected latitude gradient (see Supplementary Fig. S3A). Therefore, we conclude that the north-to-south decreasing gradient is only evident in Europe, as initially discovered by Virgili et al.^19^ These authors proposed a protective role for ocular pigmentation, which is supported by this north-to-south gradient. Additionally, Houtzagers et al.^55^ observed a significantly higher UM risk among individuals of European genetic ancestry who had green/hazel or blue/grey iris colors compared to those with brown eyes. Similarly, in those individuals with light-colored eyes, chromosome 3 and 8q aberrations had a large impact on survival, but not in those with a brown iris.^56^

The higher UM risk is potentially linked to SNP rs12913832, which serves as a significant predictor for iris color^57^ and is also associated to UM risk.^12^ In our analyses, we found that the distribution of this SNP is correlated with both OM incidence and light iris color. Importantly, Stern et al.^12^ demonstrated an association between UM risk and rs12913832 (odds ratio [OR] = 0.57, 95% confidence interval [CI] = 0.48 to 0.67, *P* = 1.88 × 10^–11^), which lost statistical significance when iris color was included as a covariate (OR = 0.76, 95% CI = 0.57 to 1.02, *P* = 0.06).

In our study, we also found a correlation between the distribution of other specific SNPs (rs1393350, rs1800407, rs3759710, rs1220592, and rs16891982) and OM incidence. However, all these SNPs are also correlated with rs12913832, complicating the differentiation of their independent effects (see Supplementary Fig. S7). Whether the correlation between OM incidence and pigmentation-related SNPs represents a true causal association, requires more in-depth investigation.

Temporal trends in OM incidence showed that a majority (80%) of countries have stable incidence, whereas some do experience a decrease or even an increase, which could likely be influenced by age-cohort effects (see the Table). Overall, the spatial distribution and trends in OM incidence in our study are similar to earlier studies (Supplementary Table S7), although precise comparison can be difficult due to several factors. First, ASRs between studies of the same population can differ, if different standard population are used. Second, variations in the coverage of study period can lead to different numbers of cases, and consequently, different ASR values. Third, not all studies use data from CI5, which could also result in a different number of cases. Nevertheless, there were a few notable differences. In Finland, we found a decrease during our study period between 1988 and 2012, whereas an earlier study^19^ found a stable trend between 1983 and 1994. In Sweden, an increasing incidence was estimated, whereas Gill et al.^22^ reported a stable incidence from 1960 to 2010, a much broader period than our study period. For Denmark, Smidt-Nielsen et al.^21^ reported an increasing incidence rate, that did not reach statistical significance in our study (see the Table), which might be explained by their wider coverage from 1947 to 2017 compared to our study period.

Unfortunately, in our study, we were unable to differentiate between UM and conjunctival melanoma cases due to unavailability of specific data regarding ocular sites. However, it is worth noting that conjunctival melanoma accounts for only 4.8% to 7% of all OM cases.^1^^,^^29^^,^^31^ Therefore, our conclusions are most likely specific for UM. Although we had a large number of cases, most African countries did not meet the requirement of a minimum number of 10 OM cases necessary for statistical analysis. The incidence of UM in the African population is already rare with a mean age of onset between 52 and 53.9.^58^^,^^59^ Despite having data of some African cancer registries, the low number of cases in Africa might be explained by a lower life expectancy (see Supplementary Fig. S5F), and the absence of comprehensive and accurate death registration systems.^60^ In order to analyze the pigmentation-related SNPs, we opted for the 1KGP dataset,^52^ where we could access a sufficient number of allele frequencies of specific SNPs from 26 populations rather than the gnomAD database^61^ with only 9 populations. Demographics and ocular pigmentation profiles of Western countries may change through time by net migration, as Eurostat data shows a net migration for 15 countries of the European Union, which peaked in 1992, decreased until 1997, and then started to rise again.^62^ Further study of migration profiles would be of interest for future studies.

In conclusion, in this comprehensive dataset of OM derived from 347 cancer registries worldwide, we estimated trends in incidence between 1988 and 2012. Overall trends remained stable and the spatial distribution showed a north-to-south decreasing latitude gradient in Europe. In addition, distribution of OM incidence correlated to blue iris color and SNP rs12913832, which is associated with both iris color^14^ and UM risk.^10^^,^^12^ We suggest that the distribution of rs12913832 can potentially explain the worldwide distribution of iris color and ocular melanoma. Further research is necessary to investigate if these correlations bear true associations.

## Supplementary Material

Supplement 1
