# Supplementary material for: Worldwide Incidence of Ocular Melanoma and Correlation With Pigmentation-Related Risk Factors
Source: Invest Ophthalmol Vis Sci. 2023 Oct 30;64(13):45. doi: 10.1167/iovs.64.13.45 (PMC10617638; doi:10.1167/iovs.64.13.45)
Supplement: Supplement 1 [file iovs-64-13-45_s001.pdf]

Table S1. Registry list and matched populations

Country	Registry	Period	Standard Population	1KG† Population
**Europe**				
Iceland	Iceland	1988-2012	European	CEU
Finland	Finland	1988-2012	European	FIN
Norway	Norway	1988-2012	European	CEU
Sweden	Sweden	1988-2007	European	CEU
Estonia	Estonia	1988-2012	European	CEU
Latvia	Latvia	1998-2012	European	CEU
Denmark	Denmark	1988-2012	European	CEU
United Kingdom	East-England	1988-2012	European	GBR
United Kingdom	East-Midlands	1993-2012	European	GBR
United Kingdom	London	1993-2012	European	GBR
United Kingdom	Merseyside and Cheshire	1988-2002	European	GBR
United Kingdom	North-East	1993-2012	European	GBR
United Kingdom	North-West	1988-2012	European	GBR
United Kingdom	Northern Ireland	1993-2012	European	GBR
United Kingdom	Oxford	1998-2007	European	GBR
United Kingdom	Scotland	1988-2012	European	GBR
United Kingdom	South-East	1993-2012	European	GBR
United Kingdom	South-West	1988-2012	European	GBR
United Kingdom	Thames	1988-2007	European	GBR
United Kingdom	Trent	1993-2007	European	GBR
United Kingdom	Wales	1988-2012	European	GBR
United Kingdom	West-Midlands	1988-2012	European	GBR
United Kingdom	Yorkshire-Humber	1988-2012	European	GBR
Lithuania	Lithuania	1993-2012	European	CEU
Ireland	Ireland	1993-2012	European	GBR
Netherlands	Netherlands	1988-2012	European	CEU
Germany	Bavaria	2008-2012	European	CEU
Germany	Brandenburg	1998-2007	European	CEU
Germany	Bremen	2003-2012	European	CEU
Germany	Eastern States	1988-1992	European	CEU
Germany	Free State of Saxony	1998-2007	European	CEU
Germany	Hamburg	1998-2012	European	CEU
Germany	Lower Saxony	2008-2012	European	CEU
Germany	Mecklenburg-Western Pomerania	1998-2007	European	CEU
Germany	Munich	1998-2012	European	CEU
Germany	North Rhine-Westphalia	1998-2012	European	CEU
Germany	Rhineland-Palatinate	2008-2012	European	CEU
Germany	Saarland	1988-2012	European	CEU
Germany	Schleswig-Holstein	2003-2012	European	CEU
Belgium	Antwerp	1998-2002	European	CEU
Belgium	Belgium	2003-2012	European	CEU
Belgium	Flanders	1993-2002	European	CEU
Belgium	Limburg	1993-1997	European	CEU
Austria	Austria	1998-2012	European	CEU
Austria	Tyrol	1988-1997	European	CEU
Austria	Vorarlberg	1993-1997	European	CEU
Switzerland	Basel	1988-2012	European	CEU
Switzerland	Fribourg	2008-2012	European	CEU
Switzerland	Geneva	1988-2012	European	CEU
Switzerland	Graubünden and Glarus	1988-2012	European	CEU
Switzerland	Neuchatel	1988-2012	European	CEU
Switzerland	St Gall-Appanzell	1988-2012	European	CEU
Switzerland	Ticino	1998-2012	European	CEU
Switzerland	Valais	1993-2012	European	CEU
Switzerland	Vaud	1988-2012	European	CEU
Switzerland	Zurich	1988-2012	European	CEU
France	Bas-Rhin	1988-2012	European	CEU
France	Calvados	1988-2012	European	CEU
France	Cote d'or	1993-1997	European	CEU
France	Doubs	1988-2012	European	CEU
France	Gironde	2008-2012	European	CEU
France	Haut-Rhin	1988-2012	European	CEU
France	Herault	1988-2012	European	CEU
France	Isere	1988-2012	European	CEU
France	Lille-Métropole	2008-2012	European	CEU
France	Limousin	2008-2012	European	CEU
France	Loire-Atlantique	1998-2012	European	CEU
France	Manche	1993-2012	European	CEU
France	Somme	1983-2012	European	CEU
France	Tarn	1988-2012	European	CEU
France	Territoire de Belfort	2008-2012	European	CEU
France	Vendée	1998-2012	European	CEU
Russia	Arkhangelsk	2008-2012	European	none
Russia	Chelyabinks	2008-2012	European	none
Russia	Karelia	2008-2012	European	none
Russia	Saint Petersburg	1993-2007	European	none
Russia	Samera	2008-2012	European	none
Belarus	Belarus	1988-2012	European	none
Poland	Cracow	1988-2007	European	none
Poland	Kielce	1998-2012	European	none
Poland	Lower Silesia	1988-2012	European	none
Poland	Lublin	2008-2012	European	none
Poland	Podkarpackie	2003-2012	European	none
Poland	Warsaw	1988-2002	European	none
Czech Republic	Czech Republic	1988-2012	European	none
Slovakia	Slovakia	1988-2012	European	none
Ukraine	Ukraine	2003-2012	European	none
Slovenia	Slovenia	1983-2012	European	none
Serbia	Serbia	1998-2007	European	none
Bulgaria	Bulgaria	1998-2012	European	none
Croatia	Croatia	1988-2012	European	none
Italy	Aosta Valley	2008-2012	European	TSI
Italy	Barletta	2008-2012	European	TSI
Italy	Bergamo	2008-2012	European	TSI
Italy	Biella	1998-2012	European	TSI
Italy	Brescia	1998-2007	European	TSI
Italy	Caserta	2008-2012	European	TSI
Italy	Catania, Messina and Enna	2003-2012	European	TSI
Italy	Catanzero	2003-2012	European	TSI
Italy	Como	2003-2012	European	TSI
Italy	Cremona	2008-2012	European	TSI
Italy	Ferrara	1988-2012	European	TSI
Italy	Florence and Prato	1988-2012	European	TSI
Italy	Friuli-Venezia Giulia	2003-2012	European	TSI
Italy	Genoa	1988-2007	European	TSI
Italy	Latina	1988-2012	European	TSI
Italy	Lecco	2003-2012	European	TSI
Italy	Liguria	1993-1997	European	TSI
Italy	Macerata Province	1988-2002	European	TSI
Italy	Mantua	2003-2012	European	TSI
Italy	Milan	1998-2012	European	TSI
Italy	Modena	1988-2012	European	TSI
Italy	Naples	1998-2012	European	TSI
Italy	North East Italy Cancer Surveillance Network	1993-2002	European	TSI
Italy	Nuoro	2003-2012	European	TSI
Italy	Palermo	2003-2012	European	TSI
Italy	Parma	1988-2012	European	TSI
Italy	Piacenza	2008-2012	European	TSI

Italy	Ragusa	1988-2012	European	TSI
Italy	Reggio Emilia	1998-2012	European	TSI
Italy	Romagna	1988-2012	European	TSI
Italy	Salerno	1998-2007	European	TSI
Italy	Sassari	1993-2012	European	TSI
Italy	Sondrio	1998-2012	European	TSI
Italy	South Lombardy	2003-2012	European	TSI
Italy	South Tyrol	2003-2012	European	TSI
Italy	Syracuse	1998-2012	European	TSI
Italy	Taranto	2008-2012	European	TSI
Italy	Trapani	2003-2007	European	TSI
Italy	Trento	2003-2012	European	TSI
Italy	Trieste	1988-1992	European	TSI
Italy	Turin	1988-2012	European	TSI
Italy	Umbria	1993-2012	European	TSI
Italy	Varese	1988-2012	European	TSI
Italy	Veneto	1988-2012	European	TSI
Spain	Albacete	1988-2012	European	IBS
Spain	Asturias	1988-2012	European	IBS
Spain	Basque Country	1988-2012	European	IBS
Spain	Canary Islands	1993-2012	European	IBS
Spain	Castellón	2008-2012	European	IBS
Spain	Ciudad Real	2003-2012	European	IBS
Spain	Cuenca	1993-2012	European	IBS
Spain	Girona	1993-2012	European	IBS
Spain	Granada	1988-2012	European	IBS
Spain	La Rioja	2003-2012	European	IBS
Spain	Mallorca	1993-2012	European	IBS
Spain	Murcia	1983-2012	European	IBS
Spain	Navarra	1988-2012	European	IBS
Spain	Tarragona	1988-2012	European	IBS
Spain	Zaragoza	1988-2002	European	IBS
Portugal	Azores	2003-2012	European	none
Portugal	Porto	1998-2002	European	none
Portugal	South Regional	1998-2002	European	none
Portugal	Vila Nova de Gaia	1993-1997	European	none
Malta	Malta	1993-2012	European	none
**Oceania**				
Australia	Northern Territories	1993-2012	Australian	none
Australia	NSW and ACT	1988-2012	Australian	none
Australia	Queensland	1993-2012	Australian	none
Australia	South Australia	1988-2012	Australian	none
Australia	Tasmania	1988-2012	Australian	none
Australia	Victoria	1988-2012	Australian	none
Australia	Western Australia	1988-2012	Australian	none
New Zealand	New Zealand	1988-2012	Australian	none
French Polynesia	French Polynesia	1998-2002	Australian	none
New Caledonia	New Caledonia	2008-2012	Australian	none
**North America**				
Canada	Canada (excl. Nunavut, Quebec and Yukon)	1988-2012	US	none
United States	California, Central Valley	1988-1992	US	none
United States	California, Los Angeles: Black	1988-1997	US	ASW
United States	California, Los Angeles: Chinese	1988-1997	US	CHS
United States	California, Los Angeles: Filipino	1988-1997	US	None
United States	California, Los Angeles: Japanese	1988-1997	US	JPT
United States	California, Los Angeles: Korean	1988-1997	US	None
United States	California, Los Angeles: White	1988-1997	US	None
United States	Lousiana, Central Region: Black	1988-1997	US	ASW
United States	Lousiana, Central Region: White	1988-1997	US	none
United States	Lousiana, New Orleans: Black	1988-1997	US	ASW
United States	Lousiana, New Orleans: White	1988-1997	US	None
United States	New Jersey: Black	1993-1997	US	ASW
United States	New Jersey: White	1993-1997	US	None
United States	New York State: Black	1993-1997	US	ASW
United States	New York State: White	1993-1997	US	None
United States	NPCR: American Indian and Alaskan Native	1998-2012	US	None
United States	NPCR: Asian and Pacific Islander	1998-2012	US	None
United States	NPCR: Black	1998-2012	US	ASW
United States	NPCR: White	1998-2012	US	None
United States	SEER (9 registries): Black	1988-1997	US	ASW
United States	SEER (9 registries): White	1988-1997	US	None
Cuba	Villa Clara	1993-2007	World	None
Puerto Rico	Puerto Rico	1993-2012	World	PUR
Jamaica	Kingston and St Andrew	2003-2012	World	None
Martinique	Martinique	1998-2012	World	None
Costa Rica	Costa Rica	1998-2012	World	None
**South America**				
Colombia	Bucaramanga	2003-2012	World	CLM
Colombia	Cali	1988-2012	World	CLM
Colombia	Manizales	2003-2012	World	CLM
Colombia	Pasto	2003-2012	World	CLM
French Guiana	French Guiana	2008-2012	World	None
Ecuador	Cuenca	2003-2012	World	None
Ecuador	Guayaquil	2008-2012	World	None
Ecuador	Loja	2008-2012	World	None
Ecuador	Manabi	2008-2012	World	None
Ecuador	Quito	1988-2012	World	None
Peru	Lima	1988-2012	World	PEL
Peru	Trujillo	1988-2002	World	PEL
Brazil	Aracaju	2003-2012	World	None
Brazil	Belem	1988-1992	World	None
Brazil	Belo Horizonte	2003-2007	World	None
Brazil	Brasilia	1998-2002	World	None
Brazil	Campinas	1993-1997	World	None
Brazil	Cuiabá	1998-2007	World	None
Brazil	Curitiba	2008-2012	World	None
Brazil	Florianopolis	2008-2012	World	None
Brazil	Fortaleza	2003-2007	World	None
Brazil	Goiania	1993-2012	World	None
Brazil	Jau	2008-2012	World	None
Brazil	Pocos de Caldas	2008-2012	World	None
Brazil	Porto Alegre	1988-1992	World	None
Brazil	São Paulo	1998-2007	World	None
Uruguay	Montevideo	1988-1997	World	None
Uruguay	Uruguay	2003-2012	World	None
Chile	Bío Bío Province	2003-2012	World	None
Chile	Concepcion	2008-2012	World	None
Chile	Region of Antofagasta	2003-2012	World	None
Chile	Valdivia	1998-2012	World	None
Argentina	Bahía Blanca	1993-2007	World	None
Argentina	Chaco	2008-2012	World	None
Argentina	Concordia	1988-1997	World	None
Argentina	Córdoba	2003-2012	World	None
Argentina	Entre Rios	2008-2012	World	None
Argentina	Mendoza	2003-2012	World	None
Argentina	Tierra del Fuego	2003-2012	World	None
**Asia**				

Turkey	Antalaya	1998-2012	World	None
Turkey	Bursa	2008-2012	World	None
Turkey	Edirne	2003-2012	World	None
Turkey	Erzurum	2008-2012	World	None
Turkey	Eskisehir	2008-2012	World	None
Turkey	Izmir	1998-2012	World	None
Turkey	Samsun	2008-2012	World	None
Turkey	Trabzon	2003-2012	World	None
Cyprus	Cyprus	1998-2012	World	None
Israel	Israel	1998-2012	World	None
Jordan	Jordanians	2008-2012	World	None
Kuwait	Kuwaiti	1998-2012	World	None
Bahrain	Bahraini	1998-2012	World	None
Qatar	Qatari	2003-2012	World	None
Saudi Arabia	Riyadh	2003-2012	World	None
Oman	Omani	1993-2012	World	None
Japan	Aichi	1998-2012	World	JPT
Japan	Fukui	1998-2012	World	JPT
Japan	Hiroshima	1988-2012	World	JPT
Japan	Miyagi	1988-2012	World	JPT
Japan	Nagasaki	1988-2012	World	JPT
Japan	Niigata	2003-2012	World	JPT
Japan	Osaka	1988-2012	World	JPT
Japan	Saga	1988-2007	World	JPT
Japan	Tochigi	2008-2012	World	JPT
Japan	Yamagata	1988-2012	World	JPT
South Korea	Busan	1998-2012	World	None
South Korea	Daegu	1993-2012	World	None
South Korea	Daejeon	1998-2012	World	None
South Korea	Dangwha	1993-1997	World	None
South Korea	Gwangju	1998-2012	World	None
South Korea	Incheon	1998-2012	World	None
South Korea	Jejudo	1998-2012	World	None
South Korea	Seoul	1993-2012	World	None
South Korea	Ulsan	1998-2012	World	None
China	Anshan	2008-2012	World	CHB
China	Beijing	1993-2007	World	CHB
China	Benxi	2008-2012	World	CHB
China	Changle	1993-1997	World	CHS
China	Cixian	1993-2012	World	CHB
China	Guangzhou	1998-2012	World	CHS
China	Guanyun	2008-2012	World	CHS
China	Haimen	2008-2012	World	CHS
China	Haining	2003-2007	World	CHS
China	Hangzhou	2008-2012	World	CHS
China	Harbin	1998-2012	World	CHB
China	Hefei	2008-2012	World	CHS
China	Hengdong	2008-2012	World	CHS
China	Hong Kong	1988-2012	World	CHS
China	Huai'an	2008-2012	World	CHS
China	Jiangmen	2008-2012	World	CHS
China	Jianhu	2008-2012	World	CHS
China	Jiashan	1993-2012	World	CHS
China	Jiaxing	2003-2012	World	CHS
China	Lianyungang	2008-2012	World	CHS
China	Linzhou	2008-2012	World	CHB
China	Liuzhou	2008-2012	World	CHS
China	Maanshan	2008-2012	World	CHS
China	Macao	2003-2007	World	CHS
China	Qidong	1988-2012	World	CHS
China	Shanghai	1988-2012	World	CHS
China	Shenyang	2008-2012	World	CHB
China	Shexian	2008-2012	World	CHS
China	Sheyang	2008-2012	World	CHS
China	Taiwan	1993-1997	World	None
China	Tianjin	1988-1997	World	CHB
China	Tongling	2008-2012	World	CHS
China	Wuhan	1993-2012	World	CHS
China	Wuxi	2008-2012	World	CHS
China	Xianju	2008-2012	World	CHS
China	Xiping	2008-2012	World	CHB

China	Yangcheng	2003-2007	World	CHB
China	Yanshi	2008-2012	World	CHB
China	Yanting	2003-2012	World	CHS
China	Yueyanglou	2008-2012	World	CHS
China	Zhongshan	1998-2012	World	CDX
China	Zhuhai	2008-2012	World	CHS
Iran	Golestan	2003-2012	World	None
Pakistan	South Karachi	1993-2002	World	PJL
India	Ahmedabad	1993-2012	World	GIH
India	Bangalore	1988-2012	World	ITU
India	Barshi	1988-1992	World	ITU
India	Barshi, Paranda and Bhum	2003-2012	World	ITU
India	Bhopal	2003-2012	World	ITU
India	Cachar	2008-2012	World	BEB
India	Chennai	1988-2012	World	ITU
India	Delhi	1988-2012	World	ITU
India	Dindigul and Ambilikkai	2003-2012	World	ITU
India	Kamrup	2008-2012	World	BEB
India	Karungapapally	1988-2007	World	ITU
India	Kollam	2008-2012	World	ITU
India	Mizarom	2003-2012	World	BEB
India	Mumbai	1988-2012	World	GIH
India	Nagpur	1993-2002	World	ITU
India	New Delhi	1998-2007	World	ITU
India	Poona	1993-2012	World	ITU
India	Sikkim	2003-2012	World	BEB
India	Tripura	2008-2012	World	BEB
India	Trivandrum	1993-2012	World	ITU
India	Wardha	2008-2012	World	ITU
Thailand	Bangkok	1993-2012	World	None
Thailand	Chiang Mai	1988-2012	World	None
Thailand	Chonburi	2003-2012	World	None
Thailand	Khon Kaen	1988-2012	World	None
Thailand	Lampang	1993-2012	World	None
Thailand	Lopburi	2008-2012	World	None
Thailand	Songkhla	1993-2012	World	None
Vietnam	Hanoi	1988-1997	World	KHV
Vietnam	Ho Chi Minh City	1993-2012	World	KHV
Philippines	Manila	1988-2012	World	None
Philippines	Rizal	1993-2012	World	None
Brunei	Darussalam	2008-2012	World	None
Malaysia	Penang	1998-2012	World	None
Malaysia	Sarawak	1998-2002	World	None
Singapore	Singapore	1988-2007	World	None
**Africa**				
Tunisia	Sousse	1998-2002	World	None
Algeria	Algiers	1993-1997	World	None
Algeria	Batna	2008-2012	World	None
Algeria	Sétif	1988-2012	World	None
Egypt	Gharbiah	1998-2002	World	None
Libya	Benghazi	2003-2007	World	None
Uganda	Kampala	1993-2012	World	LWK
Uganda	Kyadondo	1988-2012	World	LWK
Kenya	Nairobi	2008-2012	World	LWK
Malawi	Blantyre	2003-2007	World	LWK
Mali	Bamako	1988-1997	World	GWD
Zimbabwe	Harare	1988-1992	World	None
South Africa	Eastern Cape	2008-2012	World	None
South Africa	PROMEC	2003-2007	World	None

List of all included cancer registries from the CI5 volumes and CI5plus, sorted by continent. Choice for standard population and 1KG population are registred in the final two columns. Abbreviations of 1KG populations are displayed in Table S3.
† 1000 Genomes Project

Table S2. Population characteristics: ASR of Ocular Melanoma and topography by country							
Population		Ocular Melanoma			Topography		
Cohort	Period	ASR †	95% CI ‡	n	Latitude	Longitude	UV radiation
Europe	1988-2012	6.62	6.56 to 6.68	49029			
Northern Europe	1988-2012	7.33	7.23 to 7.43	21075			
Iceland §	1988-2012	7.00	5.10 to 9.35	46	65	-19	957
Finland	1988-2012	8.76	8.24 to 9.31	1084	62	26	1494
Norway §	1988-2012	9.92	9.37 to 10.49	1293	60	8	1439
Sweden	1988-2007	8.70	8.30 to 9.11	1910	60	19	1587
Estonia §	1988-2012	7.16	6.35 to 8.05	291	59	25	1781
Latvia	1998-2012	5.73	5.14 to 6.38	344	57	25	1671
Denmark §	1988-2012	11.04	10.51 to 11.58	1727	56	10	1691
United Kingdom	1988-2012	6.57	6.45 to 6.68	13062	55	-3	1576
Lithuania §	1993-2012	7.49	6.88 to 8.13	574	55	24	1801
Ireland	1993-2012	10.17	9.44 to 10.93	744	53	-8	1509
Western Europe	1988-2012	7.29	7.16 to 7.42	12957			
Netherlands §	1988-2012	9.22	8.93 to 9.52	3921	52	5	1662
Germany	1988-2012	7.22	7.01 to 7.43	4909	51	10	1812
Belgium §	1993-2012	6.04	5.67 to 6.43	1061	51	4	1645
Austria	1988-2012	6.16	5.77 to 6.57	977	48	15	1888
Switzerland §	1988-2012	5.45	5.02 to 5.91	631	47	8	2158
France	1988-2012	6.44	6.11 to 6.79	1458	46	2	1907
Eastern Europe	1988-2012	5.93	5.81 to 6.05	10382			
Russia §	1993-2012	5.48	5.07 to 5.91	673	62	105	1795
Belarus	1988-2012	7.81	7.47 to 8.16	2040	54	28	1795
Poland §	1988-2012	5.69	5.33 to 6.08	904	52	19	1749
Czech Republic	1988-2012	7.07	6.77 to 7.39	2047	50	15	1707
Slovakia §	1988-2012	8.26	7.75 to 8.8	986	49	20	1795
Ukraine	1988-2012	4.65	4.47 to 4.85	2456	48	31	1843
Slovenia §	1988-2012	6.64	5.98 to 7.35	383	46	15	2256
Serbia	1998-2007	5.71	5.11 to 6.37	357	44	21	NA
Bulgaria §	1998-2012	3.67	3.36 to 4.01	536	43	25	2331
Southern Europe	1988-2012	4.58	4.45 to 4.72	4615			
Croatia	1988-2012	6.00	5.59 to 6.44	795	45	15	1976
Italy §	1988-2012	4.86	4.66 to 5.06	2612	42	13	2444
Spain	1988-2012	3.72	3.5 to 3.96	1065	40	-4	2705
Portugal §	1993-2012	3.84	2.98 to 4.88	125	39	-8	2585
Malta	1993-2012	2.09	1.23 to 3.32	18	36	14	3091
North America	1988-2012	6.19	6.13 to 6.26	34455			
Canada §	1988-2012	7.28	7.07 to 7.50	4361	56	-106	1887
United States	1988-2012	6.18	6.11 to 6.25	29919	37	-96	2535
American Indian	1998-2012	1.85	1.37 to 2.43	58			
Asian American	1988-2012	0.96	0.81 to 1.12	163			
African American	1988-2012	0.63	0.56 to 0.70	329			
White American	1988-2012	7.13	7.05 to 7.21	29304			
Cuba §	1993-2007	1.46	0.72 to 2.67	11	22	-78	4401
Puerto Rico	1993-2012	1.05	0.80 to 1.36	63	18	-67	NA
Costa Rica §	1988-2012	1.04	0.84 to 1.29	99	10	-84	4884
Oceania	1988-2012	8.93	8.69 to 9.18	5008			
Australia §	1988-2012	8.71	8.44 to 8.98	4065	-25	134	3206
New Zealand §	1988-2012	10.18	9.54 to 10.86	941	-41	175	2487
Asia ‖	1988-2012	0.72	0.69 to 0.74	2651			
Turkey	1998-2012	1.54	1.32 to 1.79	177	39	35	2924
Cyprus	1998-2012	1.60	0.99 to 2.45	22	35	33	3439
Japan	1988-2012	0.35	0.31 to 0.40	272	36	138	2521
South Korea	1993-2012	0.82	0.73 to 0.91	364	36	128	2335
China	1988-2012	0.63	0.58 to 0.69	611	36	104	2908
Israel	1988-2012	4.07	3.76 to 4.4	761	31	35	3682
Jordan	2008-2012	1.58	1.05 to 2.28	30	31	36	4026
India	1988-2012	0.27	0.21 to 0.33	160	21	79	4514
Thailand	1988-2012	0.45	0.35 to 0.57	80	16	101	4862
Vietnam	1988-1992	0.46	0.30 to 0.67	29	14	108	4293
Philippines	1988-2012	1.17	0.70 to 1.76	72	13	122	4928
Malaysia	1998-2012	0.53	0.27 to 0.93	15	4	102	5225
Singapore	1988-2007	0.43	0.26 to 0.66	24	1	104	3979
Africa ‖	1988-2012	0.30	0.21 to 0.59	37			
Northern Africa	1988-2012	0.27	0.14 to 0.46	13			
Uganda	1988-2012	0.37	0.19 to 0.76	15	1	32	5499
Sub-Saharan Africa	1988-2012	0.34	0.21 to 0.67	24			
South America ‖	1988-2012	1.38	1.27 to 1.50	558			
Colombia	1983-2012	1.38	1.08 to 1.73	76	5	-74	5385
Ecuador	1983-2012	0.66	0.45 to 0.95	31	-2	-78	4929
Peru	1988-2012	0.97	0.67 to 1.34	35	-9	-75	5906
Brazil	1988-2012	1.95	1.73 to 2.19	299	-14	-52	4552
Uruguay	1988-2012	1.42	1.08 to 1.84	61	-33	-56	3235
Argentina	1988-2012	1.01	0.74 to 1.34	49	-38	-64	3476

Number of cases and ASR† with 95% confidence interval of ocular melanoma sorted by region and latitude throughout 1988 to 2012. ASR is generally higher in populations of European ancestry, specifically Europe, Canada, white Americans, Australia and New Zealand.
† age-standardized incidence rate; ‡ confidence interval; § selected Western country for sensitivity analysis; ‖ all continental countries are selected for sensitivity analysis

Table S3: Allele frequencies of Irisplex and UM-risk SNPs

SNP		Non-reference allele frequency							
		**rs421284**	**rs1393350**	**rs1800407**	**rs3759710**	**rs12203592**	**rs12896399**	**rs12913832**	**rs16891982**
**Loci**		*chr5:1325475*	*chr11:89277878*	*chr15:27985172*	*chr14:89955214*	*chr6:396321*	*chr14:92307319*	*chr15:28120472*	*chr5:33951588*
**Gene**		*CLPTM1L*	*TYR*	*OCA2*	*TDP1*	*IRF4*	*LOC105370627*	*HERC2*	*SLC45A2*
**Population**	**N***	**C**	**A**	**T**	**C**	**T**	**T**	**G**	**G**
ACB[1] †	192	0.64	0.04	0.01	0.31	0.02	0.06	0.08	0.09
ASW[2]	122	0.57	0.03	0.00	0.34	0.07	0.09	0.14	0.21
BEB[3]	172	0.20	0.04	0.04	0.27	0.00	0.31	0.10	0.04
CDX[4]	186	0.27	0.00	0.00	0.22	0.00	0.36	0.00	0.00
CEU[5]	198	0.43	0.24	0.08	0.07	0.16	0.56	0.77	0.98
CHB[6]	206	0.17	0.00	0.01	0.22	0.00	0.29	0.00	0.02
CHS[7]	210	0.19	0.00	0.00	0.20	0.00	0.27	0.01	0.01
CLM[8]	188	0.36	0.10	0.05	0.21	0.04	0.29	0.27	0.64
ESN[9] †	198	0.63	0.00	0.00	0.33	0.00	0.00	0.00	0.00
FIN[10]	198	0.51	0.17	0.03	0.05	0.03	0.48	0.91	0.96
GBR[11]	182	0.44	0.28	0.07	0.09	0.18	0.39	0.82	0.98
GIH[12]	206	0.20	0.11	0.02	0.26	0.00	0.34	0.08	0.09
GWD[13]	226	0.62	0.00	0.00	0.30	0.00	0.00	0.02	0.00
IBS[14]	214	0.41	0.29	0.10	0.06	0.13	0.35	0.32	0.82
ITU[15]	204	0.20	0.01	0.03	0.31	0.01	0.33	0.05	0.05
JPT[16]	208	0.13	0.00	0.00	0.26	0.00	0.47	0.00	0.00
KHV[17]	198	0.22	0.02	0.00	0.21	0.00	0.35	0.00	0.01
LWK[18]	198	0.55	0.00	0.00	0.37	0.00	0.02	0.00	0.01
MSL[19] †	170	0.58	0.00	0.00	0.37	0.00	0.01	0.00	0.01
MXL[20] †	128	0.22	0.14	0.03	0.31	0.09	0.23	0.18	0.41
PEL[21]	170	0.13	0.07	0.00	0.33	0.03	0.27	0.11	0.16
PJL[22]	192	0.16	0.07	0.04	0.27	0.02	0.30	0.10	0.06
PUR[23]	208	0.47	0.18	0.04	0.12	0.13	0.22	0.23	0.59
STU[24] †	204	0.20	0.04	0.02	0.26	0.01	0.35	0.03	0.06
TSI[25]	214	0.45	0.25	0.10	0.04	0.09	0.38	0.42	0.97
YRI[26] †	216	0.62	0.00	0.00	0.35	0.00	0.01	0.00	0.00

Non-reference allele frequencies of 8 SNPs of interest were extracted from the 1000 genomes project (1KGP). Among the 26 available populations, 20 populations could be matched with cancer registries for subsequent analysis (Table S1).

1 African Caribbean in Barbados; 2 People with African Ancestry in Southwest USA; 3 Bengali in Bangladesh; 4 Chinese Dai in Xishuangbanna, China; 5 Utah residents (CEPH) with Northern and Western European ancestry; 6 Han Chinese in Beijing, China; 7 Southern Han Chinese; 8 Colombians in Medellin, Colombia; 9 Esan in Nigeria; 10 Finnish in Finland; 11 British in England and Scotland; 12 Gujarati Indians in Houston, TX, USA; 13 Gambian in Western Division, Mandinka; 14 Iberian Populations in Spain; 15 Indian Telugu in the UK; 16 Japanese in Tokyo, Japan; 17 Kinh in Ho Chi Minh City, Vietnam; 18 Luhya in 25 Kenya; 19 Mende in Sierra Leone; 20 People with Mexican Ancestry in Los Angeles, CA, USA; 21 Peruvians in Lima, Peru; 22 Punjabi in Lahore, Pakistan; 23 Peruvians in Lima, Peru; 24 Sri Lankan Tamil in the UK; 30 Toscani in Italia; 26 Yoruba in Ibadan, Nigeria;
* N is the same for all SNPs by population; † unmatched populations

Table S4. Overview of iris color frequencies from the literature

Country	Iris color				Reference	
	Blue and grey	Green and intermediatie	Brown and hazel	N	First Author	Year
Argentina	0.07	0.51	0.42	302	Hohl[1]	2022
Australia	0.451	0.12	0.429	893	Vajdic[2]	2001
Australia	0.4840	0.287	0.229	4577	Younan[3]	2002
Weighted average of Australia	0.4786	0.2597	0.2617	5470		
Austria	0.477	0.215	0.308	853	Nischler[4]	2012
Belgium	0.396	0.241	0.363	245	Nijsten[5]	2005
Brazil	0.08	0.1733	0.7467	1594	Ruiz-Linares[6]	2014
Cameroon †	0	0	1	201	Kocnar[7]	2019
Canada	0.377	0.186	0.436	5641	Lona-Durazo[8]	2022
Chile	0.0168	0.1038	0.8894	1561	Ruiz-Linares[6]	2014
China	0	0	1	2346	Pan[9]	2018
Colombia	0.02	0.1856	0.7944	1659	Ruiz-Linares[6]	2014
Czech Republic	0.395	0.22	0.385	377	Kocnar[7]	2019
Denmark	0.787	0.144	0.069	174	Lock-Andersen[10]	1999
Estonia	0.788	0.155	0.057	579	Walsh[11]	2011
Finland	0.505	0.373	0.096	1310	Ghiasvand[12]	2019
France	0.287	0.076	0.636	616	Walsh[11]	2011
Greece †	0.125	0.029	0.846	547	Walsh[11]	2011
Iceland	0.733	0.165	0.102	2986	Sulem[13]	2007
India	0.013	0.013	0.975	79	Kocnar[7]	2019
Iran	0.01	0.0783	0.9117	3851	Hashemi[14]	2019
Ireland	0.59	0.208	0.202	1122	McGowan[15]	2015
Italy	0.292	0.116	0.592	542	Walsh[11]	2011
Mexico	0.01	0.0661	0.9239	1622	Ruiz-Linares[6]	2014
Namibia †	0	0	1	54	Kocnar[7]	2019
Netherlands	0.7	0.08	0.23	5951	Houtzagers[16]	2020
Netherlands	0.622	0.165	0.213	1214	Sulem[13]	2007
Weighted average of the Netherlands	0.6868	0.0944	0.2271	7165		
New Zealand	0.396	0.297	0.317	101	Allwood[17]	2013
Norway	0.560	0.240	0.200	523	Meyer[18]	2021
Norway	0.762	0.068	0.170	547	Walsh[11]	2011
Weighted average of Norway	0.6633	0.1521	0.1847	1070		
Peru	0	0.046	0.954	906	Ruiz-Linares[6]	2014
Poland	0.540	0.150	0.310	1093	Walsh[11]	2013
Portugal	0.052	0.214	0.734	192	Dario[19]	2015
Romania †	0.2	211	0.589	185	Kocnar[7]	2019
Slovenia	0.447	0.257	0.295	105	Kastelic[20]	2013
Spain	0.160	0.065	0.775	511	Walsh[11]	2011
Sweden	0.5	0.127	0.194	134	Kocnar[7]	2019
Turkey	0.039	0.11	0.85	127	Kocnar[7]	2019
UK	0.715	0.076	0.209	498	Walsh[11]	2011
US	0.4	0.26	0.34	200	Dembinski[21]	2014
US	0.516	0.217	0.267	3624	Tomany[22]	2003
Weighted average of the US	0.5099	0.2192	0.2708	3824		

Iris color was categorized in blue and grey, green and intermediate, and brown and hazel. A weighted average was calculated when multiple iris color frequencies were available within a population. Five out of 35 countries could not be matched with the data of both ocular melanoma incidence and SNPs of interest.

† unmatched countries; ‡ only matched with non-reference allele frequencies of SNPs of interest of corresponding population; 1 to 22 can be found in the supplementary references section on page 20.

Table S5. Overview of cancer registries for sensitivity analysis of correlation between ocular melanoma, iris color and SNPs

Population*	Lower incidence cohort†		Medium incidence cohort‡		Higher incidence cohort§	
	Registry name	ASR	Registry name	ASR	Registry name	ASR
**CEU**	Herhault (FR)	4.65	Estonia	7.16	Denmark	11.04
**TSI**	Florence and Prato (IT)	5.23	Venice (IT)	6.28	Romagna (IT)	6.70
**IBS**	Cuenca (ES)	2.52	Tarragona (ES)	4.04	Albacete (ES)	5.55
**GBR**	West-Midlands (UK)	4.25	London (UK)	5.56	Scotland (UK)	9.23
**ASW**	NPCR: Black (US)	0.50	NPCR: Black (US)	0.50	NPCR: Black (US)	0.50
**BEB**	Mizarom (IN)	0.35	Mizarom (IN)	0.35	Mizarom (IN)	0.35
**CDX**	Zhongshan (CN)	0.33	Zhongshan (CN)	0.33	Zhongshan (CN)	0.33
**CHB**	Beijing (CN)	0.41	Beijing (CN)	0.41	Beijing (CN)	0.41
**CHS**	Shanghai (CN)	0.68	Shanghai (CN)	0.68	Shanghai (CN)	0.68
**CLM**	Cali (CO)	1.52	Cali (CO)	1.52	Cali (CO)	1.52
**FIN**	Finland	8.76	Finland	8.76	Finland	8.76
**GIH**	Mumbai (IN)	0.19	Mumbai (IN)	0.19	Mumbai (IN)	0.19
**ITU**	New Delhi (IN)	0.36	New Delhi (IN)	0.36	New Delhi (IN)	0.36
**JPT**	Osaka (JP)	0.32	Osaka (JP)	0.32	Osaka (JP)	0.32
**KHV**	Hanoi (VN)	0.93	Hanoi (VN)	0.93	Hanoi (VN)	0.93
**LWK**	Kyadondo (UG)	0.44	Kyadondo (UG)	0.44	Kyadondo (UG)	0.44
**PEL**	Lima (PE)	1.02	Lima (PE)	1.02	Lima (PE)	1.02
**PJL**	South Karachi (PK)	0.88	South Karachi (PK)	0.88	South Karachi (PK)	0.88
**PUR**	Puerto Rico (PR)	1.05	Puerto Rico (PR)	1.05	Puerto Rico (PR)	1.05

The †lowest, ‡medium and §highest incidence of CEU, TSI, IBS and GBR of matched cancer registries were selected for sensitivty analysis. The displayed corresponding average ASRs of the period 1988-2012 were used in the sensitivity analysis, of which the results are displayed in table S6. Only the cancer registries differ for the European populations (CEU, TSI, IBS and GBR).
* abbreviations are displayed in Table S3.

Table S6. Sensitivity analysis of Spearman's correlation coefficient between ocular melanoma incidence, iris color frequency and non-reference allele frequency of specific SNPs

Subgroup	Iris Color			Non-reference allele frequency of SNPs							
	Blue and grey	Green and intermediatie	Brown and hazel	rs421284	rs1393350	rs1800407	rs3759710	rs12203592	rs12896399	rs12913832	rs16891982
Ocular melanoma dataset	0.55*	0.56*	-0.68**	0.43	0.71***	0.59**	-0.69**	0.83***	0.27	0.83***	0.83***
Lower incidence cohort †	0.64*	0.71**	-0.76**	0.49*	0.73***	0.60**	-0.71***	0.79***	0.27	0.82***	0.81***
Medium incidence cohort ‡	0.68**	0.71**	-0.80***	0.48*	0.73***	0.60**	-0.70***	0.79***	0.28	0.82***	0.81***
Higher incidence cohort §	0.71**	0.67**	-0.81***	0.47*	0.74***	0.62**	-0.68**	0.74***	0.28	0.82***	0.82***

A sensitivity analysis was performed on the Spearman's correlation coefficient between ASR, iris color and the non-reference allele frequency of SNPs for a lower incidence cohort †, medium incidence cohort ‡ and higher incidence cohort § (Table S5). The sensitivity analysis returned mostly similar values for all three cohorts compared to the ocular melanoma dataset. Notably, the correlation coefficients between ASR and all three iris colors increased in the lower, medium and higher incidence cohort.

* p ≤ 0.05; ** p ≤ 0.01; *** p ≤ 0.001; † ‡ § see Table S5. for the cancer registry list per subgroup.

Table S7: Summary of published reports on ocular and uveal melanoma incidence

First Author	Year	Region	Reference study: Period	Reference study: Incidence rate†	Reference study: 95% CI ‡	Reference study: No. of cases	Reference study: Trend	Our study: Period	Our study: Incidence rate†	Our study: 95% CI ‡	Our study: No. of cases	Our study: Trend
Beasley[23]	2022	Australia	1982-2014	7.6	7.3 to 7.9	4617	+/-	1988-2012	8.71	8.44 to 8.98	4065	=
Conte[24]	2018	Canada	2011-2017	5.09	4.73 to 5.44	1215	+	1988-2012	7.28	7.07 to 7.50	4361	=
Liu[25]	2013	China	1990-2005	0.6	NA	26	NA	1988-2012	0.63	0.58 to 0.69	611	=
Stang[26]	2005	Costa Rica*	1983-1997	0.74	0.20	22	NA	1988-2012	1.04	0.84 to 1.29	99	=
Stang[26]	2005	Czech Republic*	1985-1997	5.10	0.25	919	=	1988-2012	7.07	6.77 to 7.39	2047	=
Smidt-Nielsen[27]	2021	Denmark	1943-2017	13 during 2014 - 2017	NA	3344	+	1988-2012	11.04	10.51 to 11.58	1727	=
Stang[26]	2005	England*	1983-1997	4.45	0.20	1705	=	1988-2012	6.57	6.45 to 6.68	13062	=
Virgili[28]	2007	Estonia	1983-1994	7	5.8 to 8.3	129	=	1988-2012	7.16	6.35 to 8.05	291	=
Virgili[28]	2007	Finland	1983-1994	6.1	5.5 to 6.8	371	=	1988-2012	8.76	8.24 to 9.31	1084	-
Stang[26]	2005	France*	1983-1997	4.96	0.40	337	+	1988-2012	6.44	6.11 to 6.79	1458	=
Alfaar[29]	2020	Germany	2009-2015	6.41	6.21 to 6.62	3654	=	1988-2012	7.22	7.01 to 7.43	4909	=
Virgili[28]	2007	Iceland	1983-1994	6.3	3.1 to 9.5	16	=	1988-2012	7.00	5.10 to 9.35	46	=
Baily[30]	2019	Ireland	2010-2015	9.5	8.4 to 10.7	253	NA	1993-2012	10.17	9.44 to 10.93	744	-
Stang[26]	2005	Italy*	1983-1997	2.74	0.40	107	+	1988-2012	4.86	4.66 to 5.06	2612	=
Tomizuka[31]	2017	Japan	2011-2013	0.64	NA	163	NA	1988-2012	0.35	0.31 to 0.40	272	=
Virgili[28]	2007	Norway	1983-1994	8.4	7.6 to 9.2	473	=	1988-2012	9.92	9.37 to 10.49	1293	=
Nowak[32]	2022	Poland	2010-2017	8.76	4.7 to 8.5	2143	=	1988-2012	5.69	5.33 to 6.08	904	=
Aronow[33]	2017	SEER (US)	1973-2013	5.2	5.0 to 5.4	4999	=	1988-2012	6.18	6.11 to 6.25	29919	=
Stang[26]	2005	Singapore*	1983-1997	0.23	0.10	9	=	1988-2007	0.43	0.26 to 0.66	24	=
Virgili[28]	2007	Slovakia	1983-1994	6.4	5.8 to 7.1	354	=	1988-2012	8.26	7.75 to 8.8	986	=
Virgili[28]	2007	Slovenia	1983-1994	6.8	5.7 to 8.0	149	=	1988-2012	6.64	5.98 to 7.35	383	=
Park[34]	2015	South Korea	1999-2011	0.6	0.55 to 0.66	464	+	1993-2012	0.82	0.73 to 0.91	364	+
Stang[26]	2005	Spain*	1983-1997	2.21	0.36	99	=	1988-2012	3.72	3.5 to 3.96	1065	=
Gill[35]	2022	Sweden	1960-2010	5.6 to 9.6	NA	3898	=	1988-2007	8.7	8.30 to 9.11	1910	+
Stang[26]	2005	Switzerland*	1983-1997	4.10	0.70	81	=	1988-2012	5.45	5.02 to 5.91	631	=
Stang[26]	2005	The Netherlands*	1983-1997	4.64	0.72	78	=	1988-2012	9.22	8.93 to 9.52	3921	=

Incidence rates and trends of our study were mostly similar to the reported incidence rates and trends of earlier reports. Discrepancies can be influenced by difference in study period, definition of cases (uveal or ocular melanoma) and standard population.
* weighted average of male and females; † cases per million person-years; ‡ confidence interval; = stable; - decreasing; + increasing; 23 to 35 can be found in the supplementary references section on page 34.

Figure S1: Flow diagram of the systematic literature search on iris color distribution

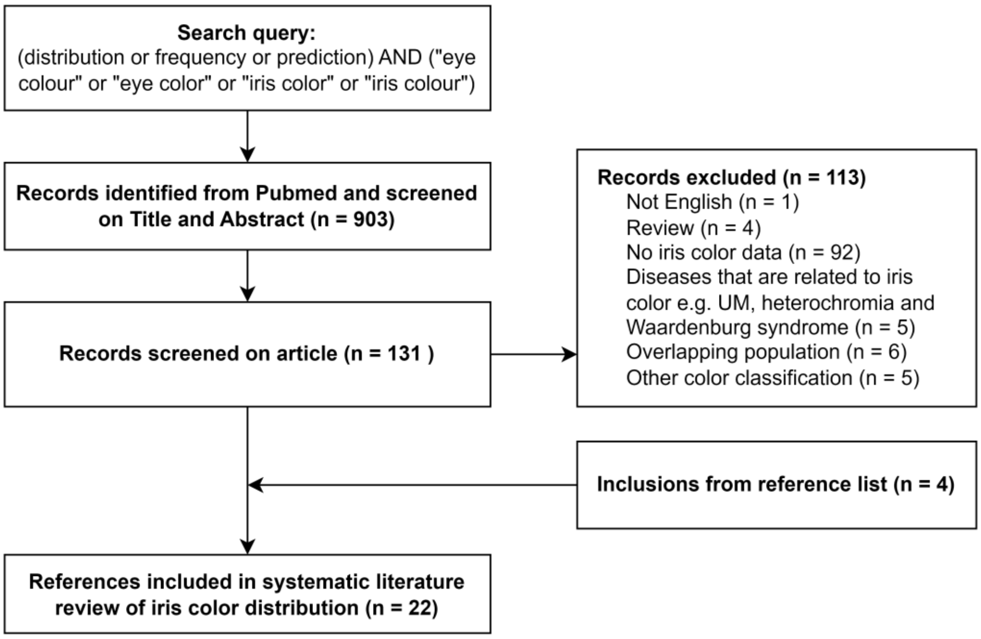

After screening 903 records on title and abstract on the Pubmed database, the full-text of 131 record were accessed. Among these, 113 records were excluded due to several reasons, mainly no iris color data (n = 92). Finally, after 4 inclusions from the reference list from screened articles, a total of 22 references were included in the literature review of iris color distribution.

Figure S2: Flow diagram of selected registries and populations

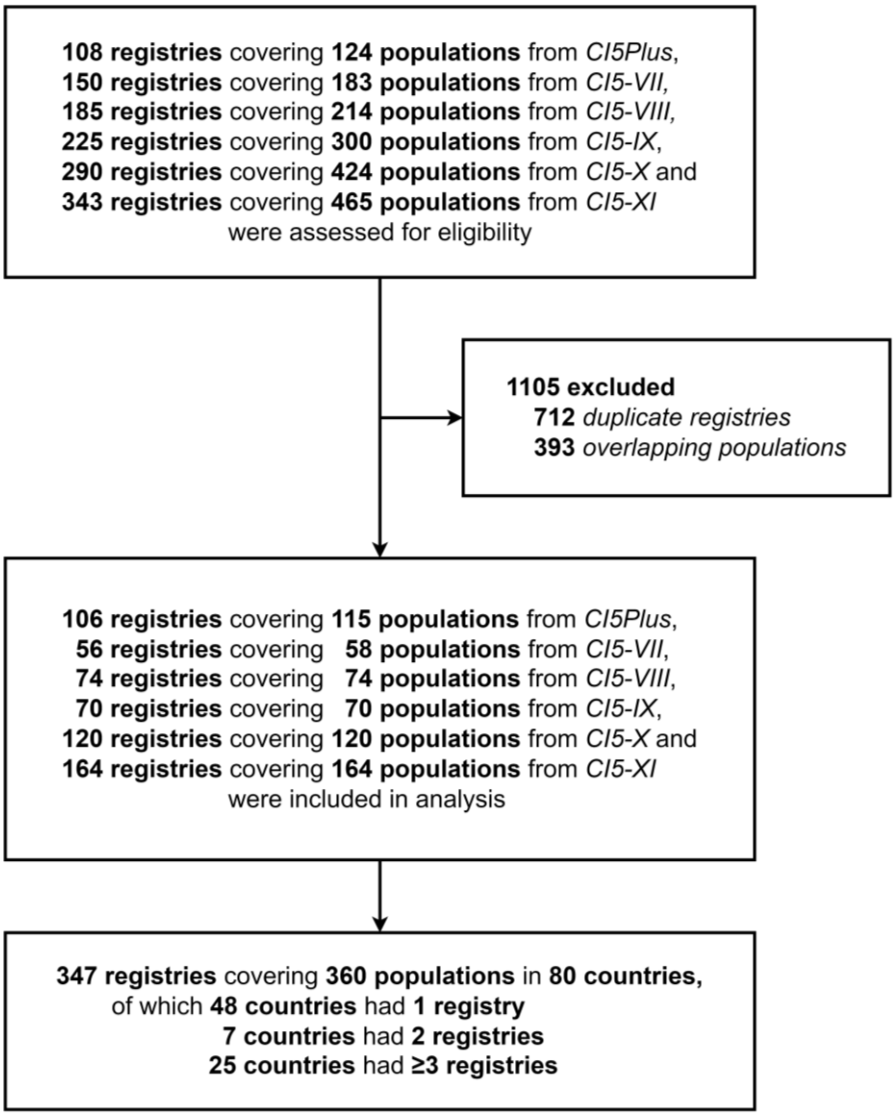

Incidence data from all cancer registries available in CI5plus and the CI5 volumes VII, VIII, IX, X and XI were extracted. Duplicate registries (n = 712) and overlapping populations (n = 393) were excluded. A total number of 347 cancer registries covering 360 populations in 80 countries from 1988 to 2012 were included. Among these, 48 countries had one registry, seven had two registries and the remaining 25 countries had three or more registries.

Figure S3: Correlation matrix of ocular melanoma incidence and topography

A

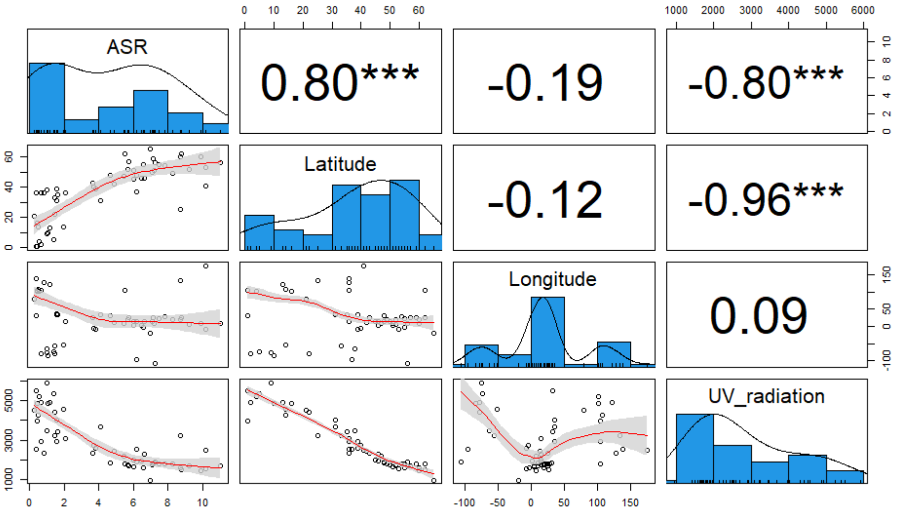

B

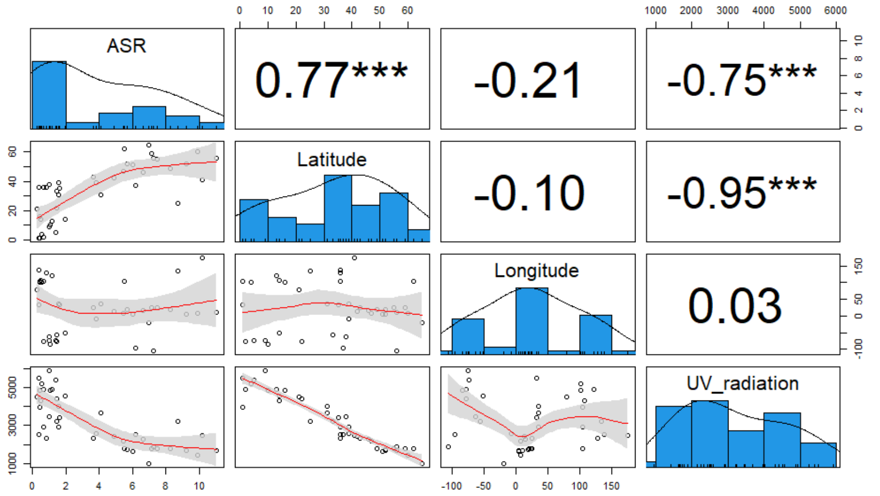

The incidence and topographic data of Table S2 were plotted into a correlation matrix (A). Spearman's rank correlation coefficients and scatterplots are displayed on the top and bottom halves of the matrix, respectively.

The scatterplot shows that Western countries typically cluster between latitudes 40 and 60, with ASR values ranging from 4 to 10. However, there are notable outliers from Asian and South American countries, often near latitude 40 or below 10, exhibiting consistently low ASR values, generally below 2. The distribution of age-adjusted incidence rates (ASR) shows a significant positive correlation to latitude ($r = 0.80$, $p \leq 0.001$). Consequently, as latitude is negatively correlated to UV-radiation ($r = -0.96$, $p \leq 0.001$), ASR is negatively correlated to UV-radiation as well ($r = -0.80$, $p \leq 0.001$).

A sensitivity analysis was performed in a 1:1 ratio of selected Western countries (Table S2, marked with §) and non-Western countries (Table S2, marked with ‖) of Asia, South America and Africa (B). The positive correlation between ASR and latitude ($r = 0.77$, $p \leq 0.001$) and the negative correlations between ASR and UV-radiation ($r = -0.75$, $p \leq 0.001$), as well as between latitude and UV-radiation ($r = -0.95$, $p \leq 0.001$), remained significant.

* $p \leq 0.05$; ** $p \leq 0.01$; *** $p \leq 0.001$

Figure S4: Age-specific incidence of ocular melanoma by gender and continent, 1988-2012

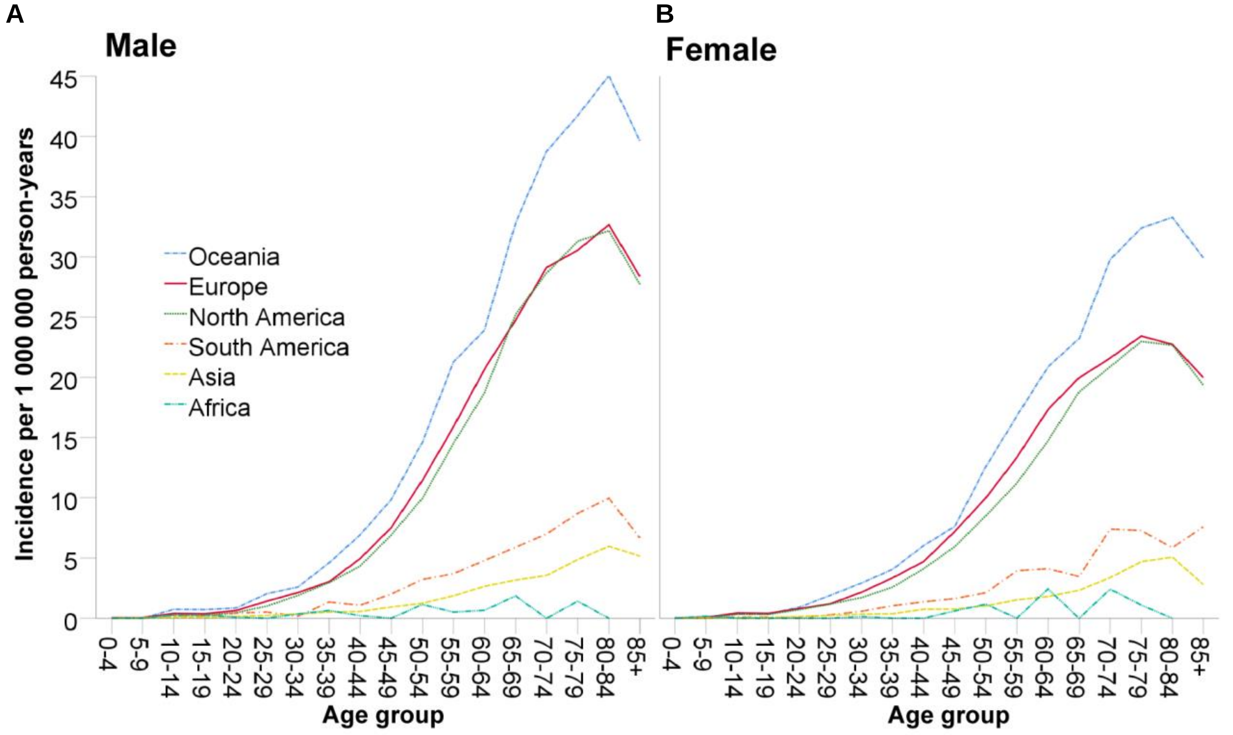

The curve of the age-specific incidence of any continent and gender follow the same pattern. In Oceania, both male and female have the same peak age of 80-84 to acquire OM (45.1 vs 32.4 cases per million person-years). Age-specific incidence is similar for Europe and North America, showing a peak at the age 80-84 (32.7 vs 32.2 cases per million person-years) for men (A), while female (B) are slightly earlier at age 75-79 (23.4 vs. 23.0). A male predominance was observed in North America, Europe and Oceania ($p \leq 0.05$).

Figure S5: Population Pyramids by continent, 1988-2012

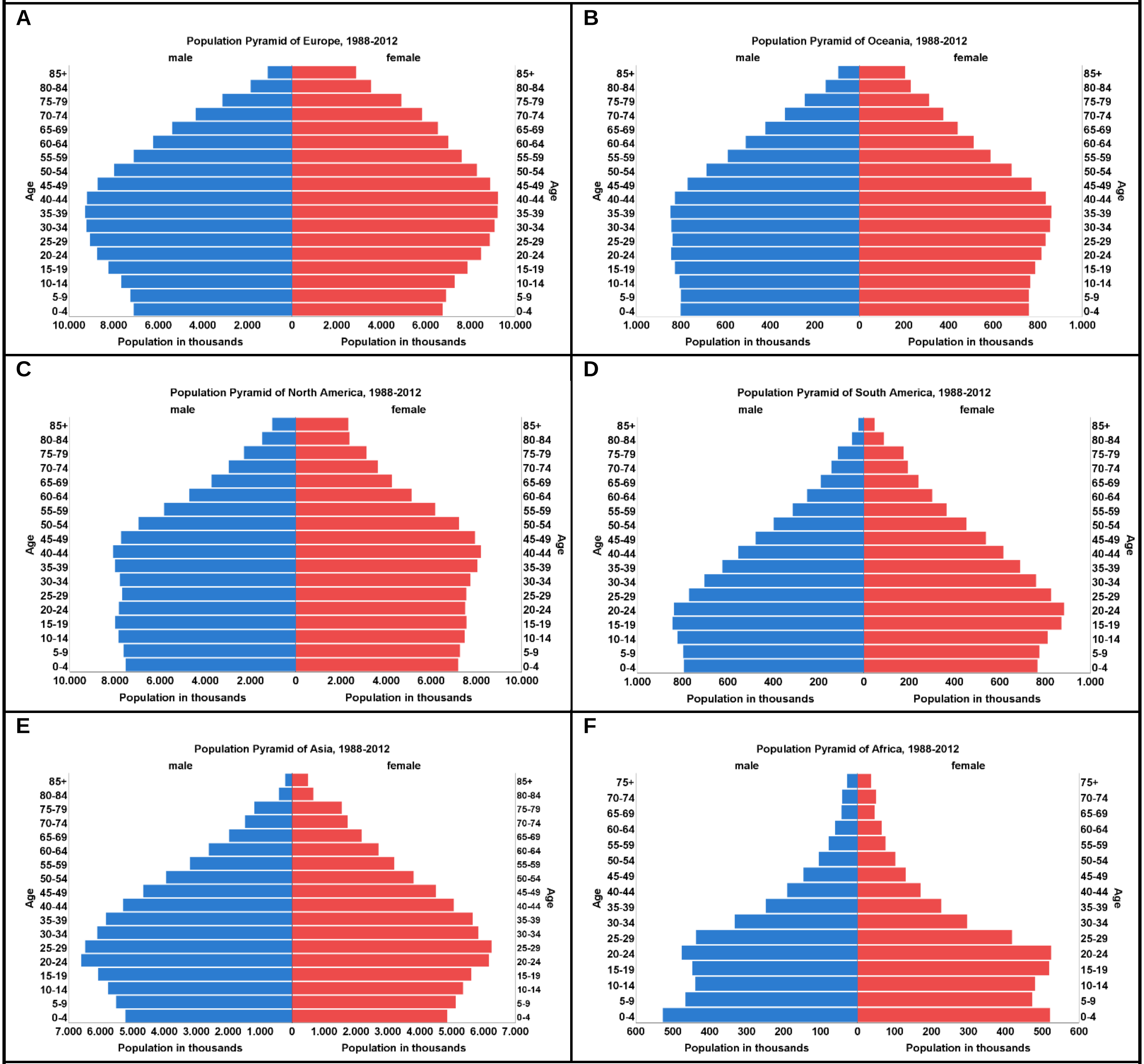

The population-at-risk of all cancer registries were aggregated by continents, resulting in corresponding population pyramids. An aging population pyramid is observed in Europe (A), Oceania (B), North America (C), while a growing population pyramid is observed in Africa (F). The population pyramids of South America (D) and Asia (E) show hallmarks of an aging as well as a growing population pyramid.

Figure S6: Worldwide distribution of allele frequency of SNP rs12913832

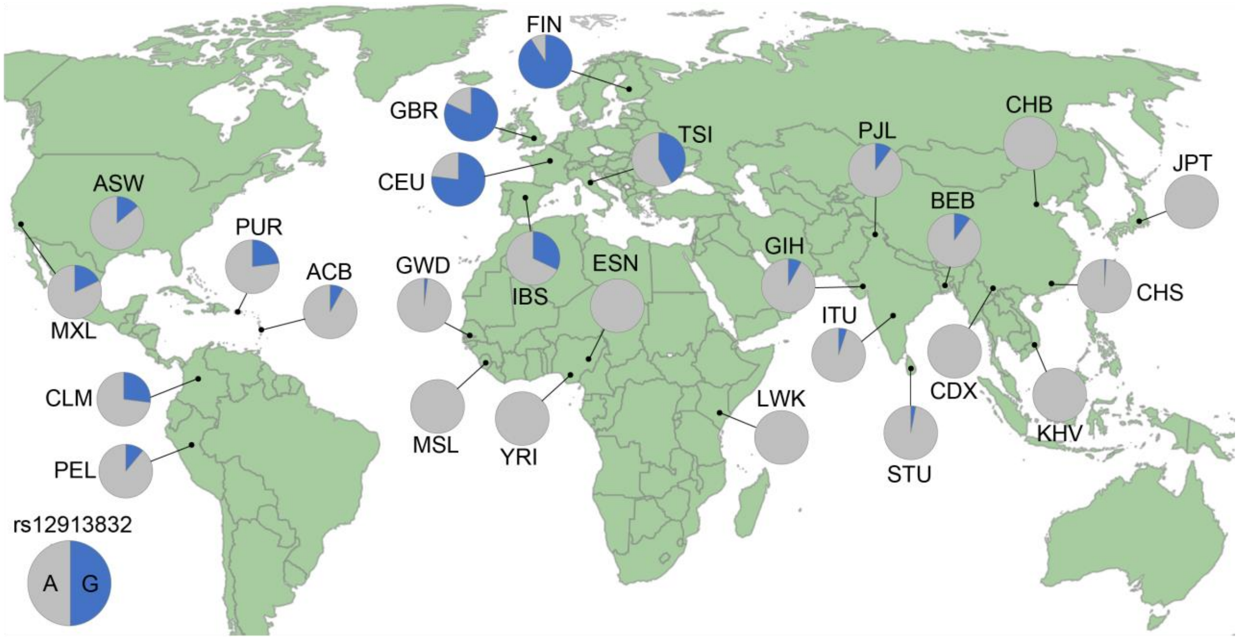

The spatial distribution of non-reference allele frequency (G) of rs12913832 – the most imporant predictor of iris color – from Table S3 has been visualized. The G allele frequency (indicated as blue in the pie charts) is highest in Europe, specifically Finnish in Finland (FIN: 0.91), British in England and Scotland (GBR: 0.82), Utah residents with Northern and Western European ancestry (CEU: 0.77), Toscani in Italy, (TSI: 0.42) and Iberian populations in Spanish (IBS: 0.32). On the contrary, the G allele frequency is zero in Chinese Dai in Xishuangbanna (CDX), Han Chinese in Beijing, China (CHB), Japanese in Tokyo, Japan (JPT), Kinh in Ho Chi Minh City, Vietnam (KHV), Luhya in Kenya (LWK), Mende in Sierra Leone (MSL) and Yoruba in Ibadan, Nigeria (YRI).

Figure S7: Correlation matrix of distribution of ocular melanoma incidence, iris color frequency and the eight SNPs of interest

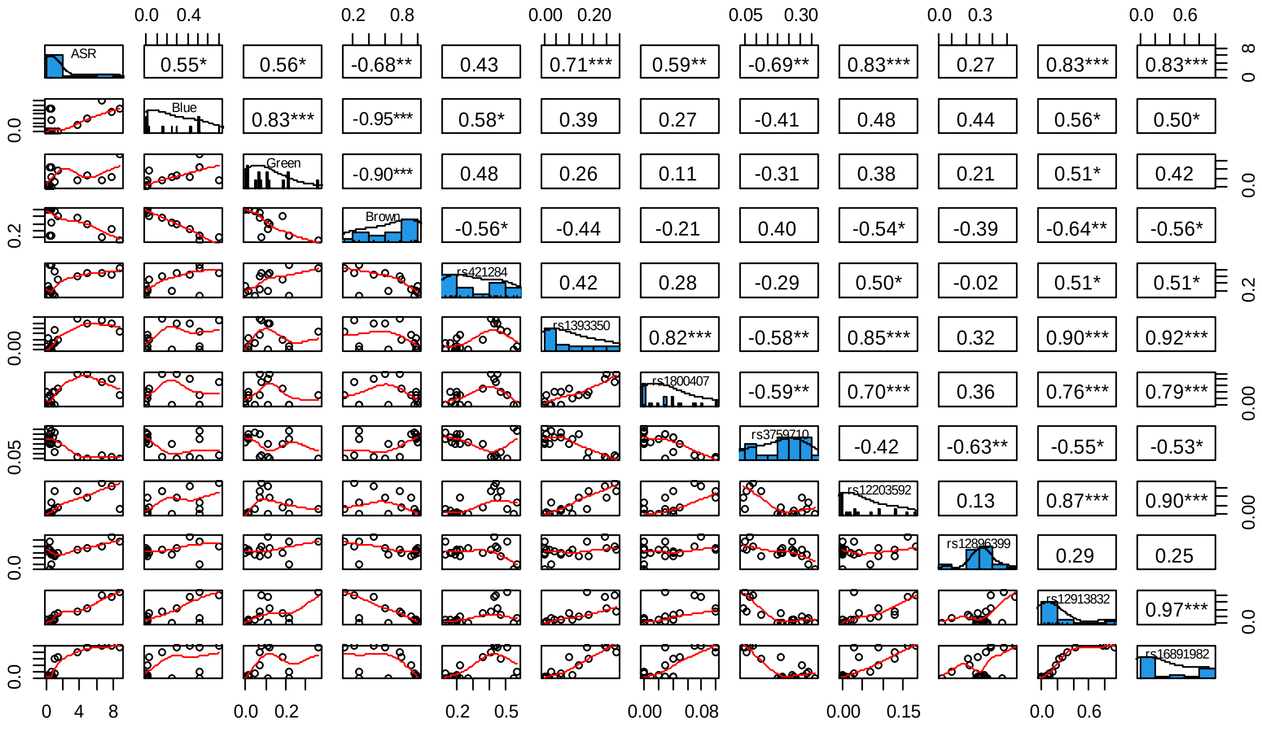

Data of age-adjusted incidence (ASR) of ocular melanoma, SNPs of interest and iris color from Table S2, S3 and S4, respectively, were plotted into a correlation matrix. Spearman's rank correlation coefficients (r) and scatterplots are displayed on the top and bottom halves of the matrix, respectively. ASR correlates negatively with brown iris color ($r = -0.68$, $p \leq 0.01$) and rs3759710 (-0.69, $p \leq 0.01$), and positively with blue (0.55, $p \leq 0.05$), rs1393350 (0.71, $p \leq 0.001$), rs1800407 (0.59, $p \leq 0.01$), rs12203592 (0.83, $p \leq 0.001$), rs12931832 (0.83, $p \leq 0.001$) and rs16891982 (0.83, $p \leq 0.001$). Among these, SNP rs12913832 – the most important predictor for iris color and associated to UM-risk according to earlier studies – correlated to the distribution of blue iris ($r = 0.56$, $p \leq 0.05$), green iris (0.51, $p \leq 0.05$) and brown iris color (-0.64, $p \leq 0.01$). Furthermore, IrisPlex SNP rs16891982 correlated to blue iris ($r = 0.50$, $p \leq 0.05$) and brown iris color (-0.56, $p \leq 0.05$), while rs12203592 correlated only to brown iris color (-0.54, $p \leq 0.05$).

* $p \leq 0.05$; ** $p \leq 0.01$; *** $p \leq 0.001$

Supplementary references

1. Hohl DM, Gonzalez R, Di Santo Meztler GP, et al. Applicability of the IrisPlex system for eye color prediction in an admixed population from Argentina. Ann Hum Genet 2022;86(6):297-327.
2. Vajdic CM, Kricker A, Giblin M, et al. Incidence of ocular melanoma in Australia from 1990 to 1998. Int J Cancer 2003;105(1):117-22.
3. Younan C, Mitchell P, Cumming RG, et al. Iris color and incident cataract and cataract surgery: the Blue Mountains Eye Study. Am J Ophthalmol 2002;134(2):273-4.
4. Nischler C, Michael R, Wintersteller C, et al. Iris color and visual functions. Graefes Arch Clin Exp Ophthalmol 2013;251(1):195-202.
5. Nijsten T, Leys C, Verbruggen K, et al. Case-control study to identify melanoma risk factors in the Belgian population: the significance of clinical examination. J Eur Acad Dermatol Venereol 2005;19(3):332-9.
6. Ruiz-Linares A, Adhikari K, Acuna-Alonzo V, et al. Admixture in Latin America: geographic structure, phenotypic diversity and self-perception of ancestry based on 7,342 individuals. PLoS Genet 2014;10(9):e1004572.
7. Kocnar T, Saribay SA, Kleisner K. Perceived attractiveness of Czech faces across 10 cultures: Associations with sexual shape dimorphism, averageness, fluctuating asymmetry, and eye color. PLoS One 2019;14(11):e0225549.
8. Lona-Durazo F, Thakur R, Pairo-Castineira E, et al. Investigating the genetic architecture of eye colour in a Canadian cohort. iScience 2022;25(6):104485.
9. Pan CW, Qiu QX, Qian DJ, et al. Iris colour in relation to myopia among Chinese school-aged children. Ophthalmic Physiol Opt 2018;38(1):48-55.
10. Lock-Andersen J, Drzewiecki KT, Wulf HC. Eye and hair colour, skin type and constitutive skin pigmentation as risk factors for basal cell carcinoma and cutaneous malignant melanoma. A Danish case-control study. Acta Derm Venereol 1999;79(1):74-80.
11. Walsh S, Liu F, Wollstein A, et al. The HIrisPlex system for simultaneous prediction of hair and eye colour from DNA. Forensic Sci Int Genet 2013;7(1):98-115.
12. Ghiasvand R, Robsahm TE, Green AC, et al. Association of Phenotypic Characteristics and UV Radiation Exposure With Risk of Melanoma on Different Body Sites. JAMA Dermatol 2019;155(1):39-49.
13. Sulem P, Gudbjartsson DF, Stacey SN, et al. Genetic determinants of hair, eye and skin pigmentation in Europeans. Nat Genet 2007;39(12):1443-52.
14. Hashemi H, Pakzad R, Yekta A, et al. Distribution of iris color and its association with ocular diseases in a rural population of Iran. J Curr Ophthalmol 2019;31(3):312-8.
15. McGowan A, Silvestri G, Moore E, et al. Retinal vascular caliber, iris color, and age-related macular degeneration in the Irish Nun Eye Study. Invest Ophthalmol Vis Sci 2014;56(1):382-7.
16. Houtzagers LE, Wierenga APA, Ruys AAM, et al. Iris Colour and the Risk of Developing Uveal Melanoma. Int J Mol Sci 2020;21(19).
17. Allwood JS, Harbison S. SNP model development for the prediction of eye colour in New Zealand. Forensic Sci Int Genet 2013;7(4):444-52.
18. Meyer OS, Salvo NM, Kjaerbye A, et al. Prediction of Eye Colour in Scandinavians Using the EyeColour 11 (EC11) SNP Set. Genes (Basel) 2021;12(6).
19. Dario P, Mourino H, Oliveira AR, et al. Assessment of IrisPlex-based multiplex for eye and skin color prediction with application to a Portuguese population. Int J Legal Med 2015;129(6):1191-200.
20. Kastelic V, Pospiech E, Draus-Barini J, et al. Prediction of eye color in the Slovenian population using the IrisPlex SNPs. Croat Med J 2013;54(4):381-6.
21. Dembinski GM, Picard CJ. Evaluation of the IrisPlex DNA-based eye color prediction assay in a United States population. Forensic Sci Int Genet 2014;9:111-7.
22. Tomany SC, Klein R, Klein BE, Beaver Dam Eye S. The relationship between iris color, hair color, and skin sun sensitivity and the 10-year incidence of age-related maculopathy: the Beaver Dam Eye Study. Ophthalmology 2003;110(8):1526-33.
23. Beasley AB, Preen DB, McLenachan S, et al. Incidence and mortality of uveal melanoma in Australia (1982-2014). Br J Ophthalmol 2023;107(3):406-11.
24. Conte S, Lagace F, Ghazawi FM, et al. Uveal melanoma incidence trends in Canada: 1992-2010 vs. 2011-2017. Front Med (Lausanne) 2022;9:1001799.
25. Liu YC, Tsai CC, Lee FL, et al. Mortality from uveal melanoma treated by enucleation --a 16-year survey in Taiwan. Acta Ophthalmol 2013;91(7):e583-4.
26. Stang A, Parkin DM, Ferlay J, Jockel KH. International uveal melanoma incidence trends in view of a decreasing proportion of morphological verification. Int J Cancer 2005;114(1):114-23.
27. Smidt-Nielsen I, Bagger M, Heegaard S, et al. Posterior uveal melanoma incidence and survival by AJCC tumour size in a 70-year nationwide cohort. Acta Ophthalmol 2021;99(8):e1474-e82.
28. Virgili G, Gatta G, Ciccolallo L, et al. Incidence of uveal melanoma in Europe. Ophthalmology 2007;114(12):2309-15.
29. Alfaar AS, Saad A, Wiedemann P, Rehak M. The epidemiology of uveal melanoma in Germany: a nationwide report of incidence and survival between 2009 and 2015. Graefes Arch Clin Exp Ophthalmol 2021.
30. Baily C, O'Neill V, Dunne M, et al. Uveal Melanoma in Ireland. Ocul Oncol Pathol 2019;5(3):195-204.
31. Tomizuka T, Namikawa K, Higashi T. Characteristics of melanoma in Japan: a nationwide registry analysis 2011-2013. Melanoma Res 2017;27(5):492-7.
32. Nowak MS, Romanowska-Dixon B, Grabska-Liberek I, Zurek M. Incidence and survival of ocular melanoma in National Cancer Registry of Poland in 2010-2017. Adv Clin Exp Med 2022;31(6):615-21.
33. Aronow ME, Topham AK, Singh AD. Uveal Melanoma: 5-Year Update on Incidence, Treatment, and Survival (SEER 1973-2013). Ocul Oncol Pathol 2018;4(3):145-51.
34. Park SJ, Oh CM, Kim BW, et al. Nationwide Incidence of Ocular Melanoma in South Korea by Using the National Cancer Registry Database (1999-2011). Invest Ophthalmol Vis Sci 2015;56(8):4719-24.
35. Gill V, Herrspiegel C, Sabazade S, et al. Trends in Uveal Melanoma Presentation and Survival During Five Decades: A Nationwide Survey of 3898 Swedish Patients. Front Med (Lausanne) 2022;9:926034.
